# Spatial transcriptomics reveals the molecular signatures of prodromal and advanced α-synucleinopathy

**DOI:** 10.1016/j.isci.2026.114845

**Published:** 2026-01-29

**Authors:** Lin Lin, Nanna M. Jensen, Alberto Delaidelli, Sara A. Ferreira, Fatemeh Yarmahmoudi, Poul H. Sorensen, Marina Romero-Ramos, Poul H. Jensen, Ian R. Mackenzie, Jens R. Nyengaard, Asad Jan

**Affiliations:** 1 Department of Biomedicine, Aarhus University, Høegh-Guldbergs Gade 10, 8000 Aarhus, Denmark; 2 Danish Research Institute of Translational Neuroscience (DANDRITE), Aarhus University, Høegh-Guldbergs Gade 10, Aarhus C, 8000 Aarhus, Denmark; 3 Department of Molecular Oncology, British Columbia Cancer Research Centre, Vancouver, BC V5Z 1L3, Canada; 4 Department of Pathology, Mass General Brigham, Harvard Medical School, Boston, MA, USA; 5 Core Center for Molecular Morphology, Section for Stereology and Microscopy, Department of Clinical Medicine, Aarhus University, Aarhus N, 8200 Aarhus, Denmark; 6 Department of Pathology and Laboratory Medicine, University of British Columbia, 2211 Wesbrook Mall, Vancouver, BC V6T 2B5, Canada

**Keywords:** Model organism, Molecular neuroscience, Protein structure aspects, Transcriptomics

## Abstract

A pathological role of α-synuclein (aSyn) aggregation in the etiology of Parkinson disease (PD) is well established. Here, we applied spatial transcriptomics (ST) on brain sections derived from a rodent mouse model of α-synucleinopathy (transgenic M83^+/+^ line). Our ST data revealed that induction of aSyn pathology in the brainstem of rodents triggered upregulation of pathways controlling energy metabolism. At a later stage, characterized by movement disability, the ST data indicated a drastic downregulation of mitochondrial metabolic pathways along with perturbed expression of mRNA translation machinery. Furthermore, analyses of microarrays datasets derived from 4 independent cohorts of PD patients led to the identification of aberrant osteopontin (SPP1) signaling and increased expression of CREB-binding protein as consistent markers associated with the progression of aSyn pathology in the rodent model and in PD brains. We anticipate that our findings hold promise for biomarker discovery and/or mechanism-based therapies in PD and related neurodegenerative disorders.

## Highlights

• Induction of α-Synuclein pathology in rodent brain stimulates mitochondrial metabolism
• Declining energy metabolism and mRNA translation herald the onset of motor defects
• Aberrant ROCK2 and SPP1 signaling reflect progressive tissue damage
• Increased CREBBP expression is a unique marker of advanced α-synuclein pathology

## Graphical abstract

**Figure:**
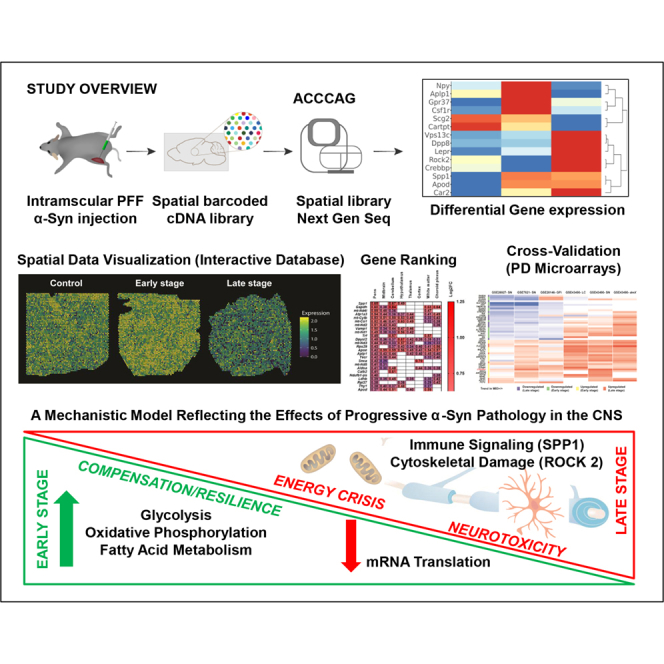

## Introduction

Cellular alpha-synuclein (aSyn; gene symbol *SNCA*) pathology in the nervous system is a hallmark feature of idiopathic Parkinson disease (PD) and related disorders termed synucleinopathies.^1^^,^^2^ Under physiological conditions, aSyn protein has been suggested to facilitate the assembly of synaptic components during vesicle release and as a chaperone.^3^^,^^4^ However, in disease states, misfolded and aggregated forms of aSyn accumulate in the nervous system as Lewy body (LB)-related aSyn pathology.^5^^,^^6^ Several studies implicate that the process of aSyn aggregation adversely affects neuronal survival by triggering impairments in mitochondrial function, endoplasmic reticulum stress, defects in protein sorting and autophagy, perturbed redox homeostasis, and neuroinflammation.^1^^,^^4^ The advent of high-content spatial omics technologies (proteomics and transcriptomics) offers unprecedented opportunities for unraveling the molecular basis of neuronal dysfunction caused by progressive aSyn pathology in PD, and for the discovery of molecular signatures indicating the progression of disease.

Seminal neuropathological studies by Braak and colleagues, and corroborated by others, suggest that pathological aSyn deposition in caudal brainstem appears long before the affection of the midbrain substantia nigra—*pars compacta* (SN*pc*).^7^^,^^8^^,^^9^ In particular, the neuronal populations within the brainstem reticular formation (BRF, in pons and medulla), dorsal motor nucleus of the vagus (dmX) and locus coeruleus (LC), bear the brunt of cellular aSyn pathology in prodromal PD (i.e., LB Braak stages I-II).^7^^,^^8^^,^^9^ In a mouse model of synucleinopathy (M83 line, *Prnp-SNCA∗A53T*, overexpressing the aggregation-prone human mutant A53T),^10^ we and others have shown that exogenous delivery of pre-formed fibrillar (PFF) aggregates of aSyn through intramuscular route reproducibly induces *de novo* aSyn aggregation in brainstem.^11^^,^^12^^,^^13^^,^^14^^,^^15^ In particular, this animal model exhibits a prodromal phase during which peripherally induced aSyn aggregation is predominantly localized within the paramedian nuclei of BRF (pontine gigantocellular nuclei, GRN, and mesencephalic periaqueductal gray, PAG), while the gross motor performance of the animals is relatively intact.^12^^,^^13^^,^^16^ Notably, both the GRN and the PAG harbor substantial pathological aSyn accumulation in PD with implications for motor and non-motor symptomatology such as pain and sleep disorder.^7^^,^^8^^,^^17^ Despite the relative lack of gross movement disability, some studies indicate that the early phase of aSyn pathology in this animal model is associated with subtle neurological dysfunction, for instance, mild-to-moderate degree of hindlimb clasping,^13^ alterations in the patterns of brain activity,^18^ defects in the nerve conduction,^12^ and pain-related behaviors.^12^ Subsequently, progressive aSyn pathology in the nuclei of somatomotor control (i.e., spinal motor neurons, red nucleus, vestibular nuclei, cerebellar nuclei, and motor cortex) eventually leads to deterioration in movement coordination and hindlimb paresis, which culminate in reduced survival.^12^^,^^13^^,^^16^

In order to decipher the cellular response to the early and progressive/symptomatic phase of aSyn pathology, we harnessed the utility of spatial transcriptomics (ST) to obtain spatially resolved gene expression profiles in the brains of this rodent model. In these analyses, we found that the prodromal/early phase of aSyn pathology was associated with enhanced expression of transcripts comprising ATP metabolic pathways in the disease-affected brain regions (i.e., glycolysis, oxidative phosphorylation, and fatty acid metabolism). In contrast, the symptomatic/late phase was characterized by a profound decline in these metabolic pathways, with concomitant perturbations in the expression of ribosomal mRNA translation machinery and microtubule transport, and compounded by inflammatory response. Crucially, we cross-validated several of the candidate transcriptomic profiles from the rodent ST dataset in 4 independent microarray datasets containing gene expression profiles in PD SN, LC, GPi (striatum, globus pallidus interna), and *dmX*. Among others, these extensive analyses led to the identification of increased osteopontin/secreted phosphoprotein (SPP1) signaling and increased expression of CREB-binding protein (CREBBP/CBP) as putative molecular signatures of progressive aSyn pathology. The methodological context and significance of these findings are further elaborated below.

## Results

### Intramuscular murine PFF aSyn delivery induced progressive aSyn aggregation in the GRN and PAG of M83^+/+^ mice

**Figure 1:**
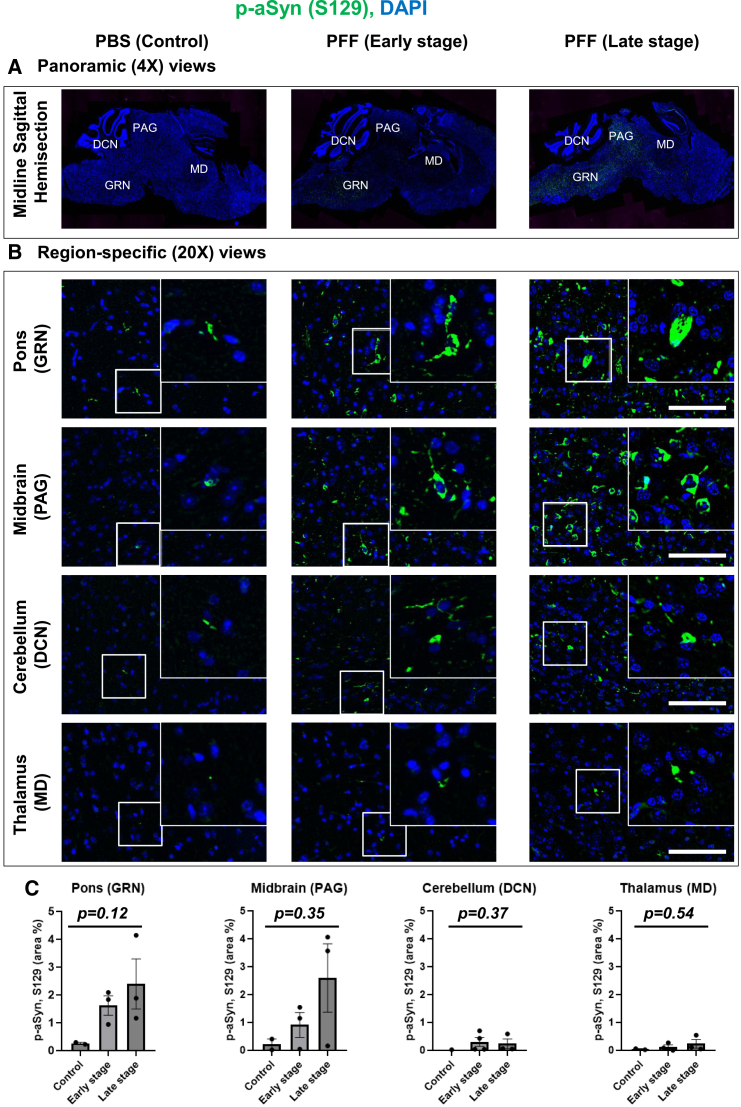
Immunofluorescence (IF) detection of phosphorylated alpha-synuclein (*p*-aSyn, S129) in brains of M83^+/+^ mice (A) Representative low-magnification (4×) panoramic images of sagittal brain sections from controls (PBS, days post injection, DPI-75) and PFF aSyn-injected M83^+/+^ mice (Early stage, DPI-45; and Late stage, DPI-75). The labels in CAPITAL LETTERS indicate specific brain regions examined for the IF analyses (in B). (B) Representative (20×) images showing *p*-aSyn (S129) IF in the gigantocellular nuclei (GRN, in pons), periaqueductal gray (PAG, in midbrain), deep cerebellar nuclei (DCN, in cerebellum) and mediodorsal nuclei (MD, in thalamus). The insets show 63× magnified views from the regions. Scale bar, 50 μm. (C) Bar graphs depicting quantification of *p*-aSyn (S129) IF in GRN, PAG, DCN, and DM of the experimental cohorts, as indicated. Error bars depict mean IF intensity ±SD as % of total area (PBS, *n* = 2; DPI-45, *n* = 3; and DPI-75, *n* = 3; see STAR Methods). Statistics in (C): Kruskal-Wallis ANOVA (*p*-values on graphs), Dunn multiple comparisons (non-significant).

In this study, we applied ST to sagittal brain sections obtained from the M83^+/+^ mice, following intramuscular delivery of murine PFF aSyn at: i) an early prodromal stage with visible lack of gross motor phenotype/weakness (ES, days post injection: DPI-45)^13^^,^^14^ and ii) a late stage with movement disability (LS, DPI-75).^14^^,^^15^ We chose to perform the ST analyses using sagittal brain sections since it allowed us to capture the transcriptomics profiles associated with early aSyn pathology in the paramedian brain regions of interest (GRN and PAG), which are affected in the prodromal phase in this animal model.^12^^,^^13^^,^^15^^,^^16^ Moreover, we could also assess the effects of aSyn aggregation in the large tracts of white matter in this model,^13^ which is a feature reminiscent of Lewy-related aSyn pathology in PD and related synucleinopathies.^5^^,^^17^ Lastly, a larger caudo-rostral “snapshot” of the whole brain can be obtained in one section (Figure 1A), which is not possible in region-limited coronal sections. In line with the previous observations,^12^^,^^13^^,^^15^^,^^16^ we detected progressive accumulation of aggregated aSyn in caudal brainstem as indicated by immunofluorescence (IF) of phosphorylated aSyn (p-aSyn, S129), which is a widely used biochemical and neuropathological marker of aSyn aggregation.^19^^,^^20^ In particular, substantial aSyn aggregation was detected in pons (GRN) and midbrain (PAG) at both the ES and the LS, and to a lesser extent in deep cerebellar nuclei (DCN) and thalamic (mediodorsal, MD) nuclei (Figures 1B and 1C). However, due to a small cohort size (controls: PBS, *n* = 2; ES, *n* = 3; and LS, *n* = 3), statistical significance was not achieved (Figure 1C).

### The ST profiles clustered in-register with distinct neuroanatomical annotations and the stage of aSyn pathology in brains of M83^+/+^ mice

**Figure 2:**
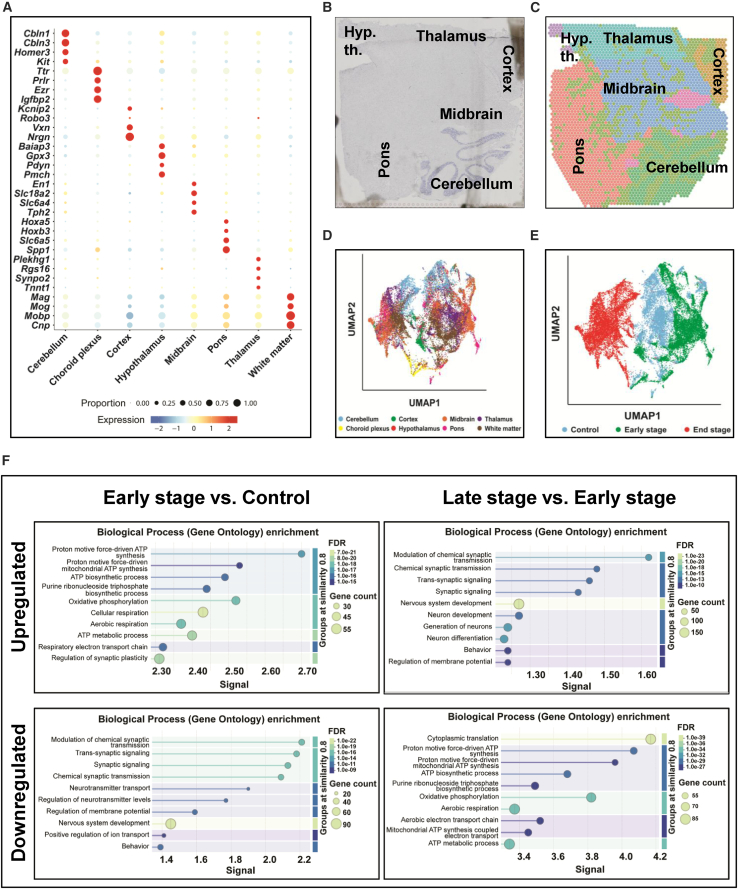
Overview of ST annotations and differentially expressed pathways in relation to the stage of aSyn pathology in brains of M83^+/+^ mice (A) Bubble plot depicting the regionally enriched markers used for delineating 8 ST annotations in sagittal brain sections from M83^+/+^ mice. (B and C) Representative low-magnification (4×) panoramic images of a sagittal brain section showing gray matter regions (in B, hematoxylin counterstain) and corresponding spatial map (in C). (D) UMAP plot depicting distinct clustering of ST profiles in annotations, which additionally includes white matter and choroid plexus based on the abundance of regional markers (in 2A; also see STAR Methods). (E) UMAP plot depicting distinct clustering of ST profiles in relation to the stage of aSyn pathology in brains of M83^+/+^ mice. (F) Dot plots depicting gene ontology (GO) of differentially expressed ST profiles in brains of M83^+/+^ mice. The plots were generated in the STRING protein interactions database. Also see Figure S1B, depicting heatmaps for all HALLMARK gene sets by disease stage and in individual regions.

For the quantitative analyses of ST profiles, we employed a pipeline for annotating the spatially barcoded cDNA sequencing data onto histological map of the sagittal brain sections from M83^+/+^ cohorts (see STAR Methods). For this purpose, we assigned each capture spot to distinct brain regions based on the expression of regionally enriched transcripts in mouse brain^21^ and histological landmarks from Mouse Brain Atlas (namely, pons, midbrain, cerebellum, thalamus, hypothalamus, and cortex including hippocampal formation; Figures 2B and 2C).^22^^,^^23^ Furthermore, to avoid ambiguity between the gray vs. non-gray matter, the ST annotations were also adapted to account for large white matter tracts and cerebral ventricular system (choroid plexus). In brief, the resultant 8 ST annotation/region profiles exhibited regional enrichment of unique transcripts reflecting synaptic markers in gray matter (e.g., *Homer3* in cerebellum, *Tph2* in midbrain, and *Slc6a5* in pons), structural components in the white matter (e.g., *Mobp*), and choroid plexus in the cerebral ventricles (e.g., *Ttr*); Figure 2A. This is further illustrated in Figure 2B (histological image of a brain section) and Figure 2C (with corresponding color-coded spatial map); also see Figure 4A for additional examples.

Admittedly, there were limitations to conclusively differentiate every spatial element (capture spot) into the gray and non-gray matter categories at the microscopic detail, especially in pons and midbrain (Figure 2A; compare pons with cortex or choroid plexus). This is likely due to (i) the applied technology (10× Genomics Visium v1) lacks the single-cell resolution (each spot in the capture area is 55 μm in diameter with an inter-spot center-to-center distance of 100 μm) and/or (ii) the two regions (pons and midbrain) contain dense clusters of white matter in the neuropil and are traversed by large sensorimotor tracts of spinal cord.^23^ Thus, our ST annotations for the 8 regional profiles (Figures 2A–2C) reflect gross anatomical organization of brain (and not single-cell, microscopic organization distinguishing neuronal and glial cells). With this limitation in perspective, below we describe the salient findings from the ST study in this rodent model of progressive aSyn pathology.

### ST unveiled unique molecular signatures associated with the early and late stages of aSyn pathology in brains of M83^+/+^ mice

**Figure 3:**
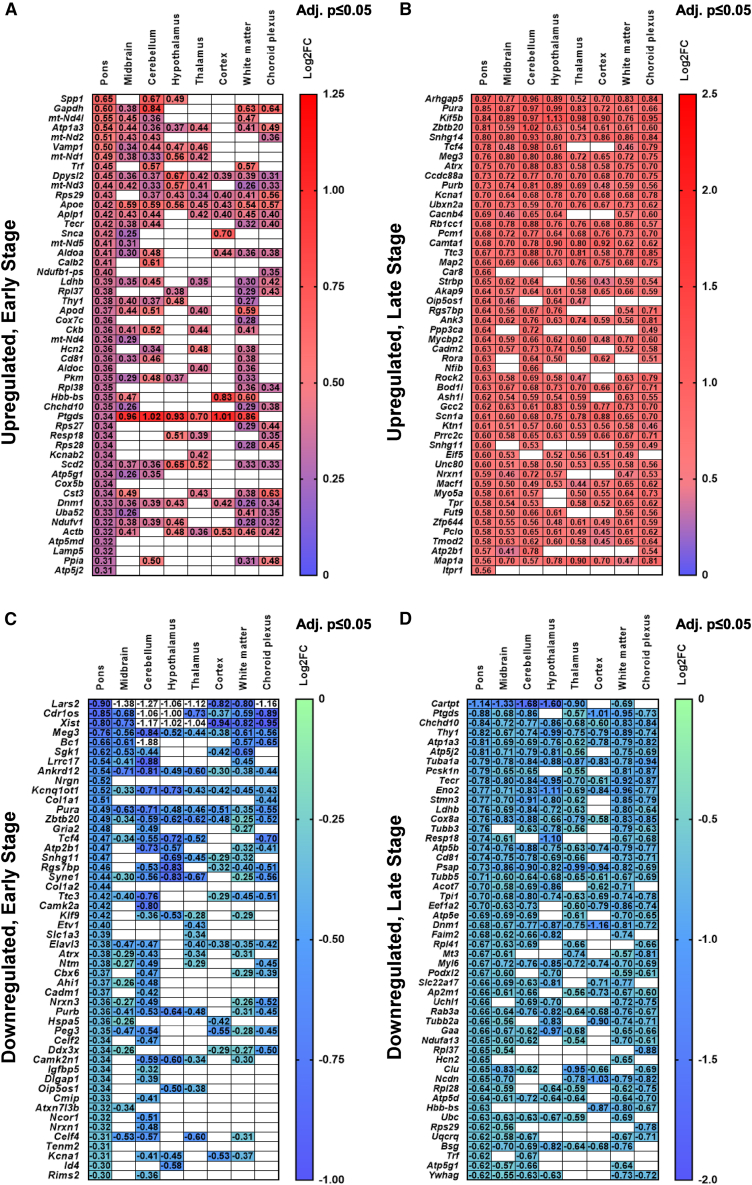
Heatmaps depicting the expression of select 50 genes across ST annotations in relation to the stage of aSyn pathology in brains of M83^+/+^ mice (A–D) Gene expression profiles indicated by the Log2FC values, which were upregulated at early stage (in A), upregulated at late stage (in B), downregulated at early stage (in C), and downregulated at late stage (in D) with adjusted *p*-value ≤0.05. Also see S2 for the expression of top 10 transcripts unique to each ST annotation. Also see Tables S2–S5 for complete lists with Log2FC and adjusted *p*-value for all upregulated and downregulated transcripts in relation to the stage of aSyn pathology in brains of M83^+/+^ mice.

Altogether, we identified 16,833 unique protein coding transcripts in the ST data, which clustered distinctively within the anatomical annotations/regions (Figure 2D, UMAP plots) and between the experimental cohorts (Figure 2E, UMAP plots). Next, we applied cut-off criteria (Log2 fold change ≥±0.25; adjusted *p* ≤ 0.05) to prioritize significantly enriched transcripts, which were upregulated or downregulated by the stage of aSyn pathology. This approach led to the identification of global transcriptomics profiles, i.e., ES: 961 upregulated, 241 downregulated and LS: 669 upregulated, 1,465 downregulated (for region-specific breakdown: see Figure S1A). Majority of the transcripts were shared among the annotations as illustrated in Figures 3A–3D, while a limited number of unique profiles were also found (Figures S2A–S2D). Complete gene ranking information of the transcripts that fulfilled the cut-off criteria are listed for each region with respective LogFC and adjusted *p*-value in supplementary excel files (Tables S2, S3, S4, and S5).

**Figure 4:**
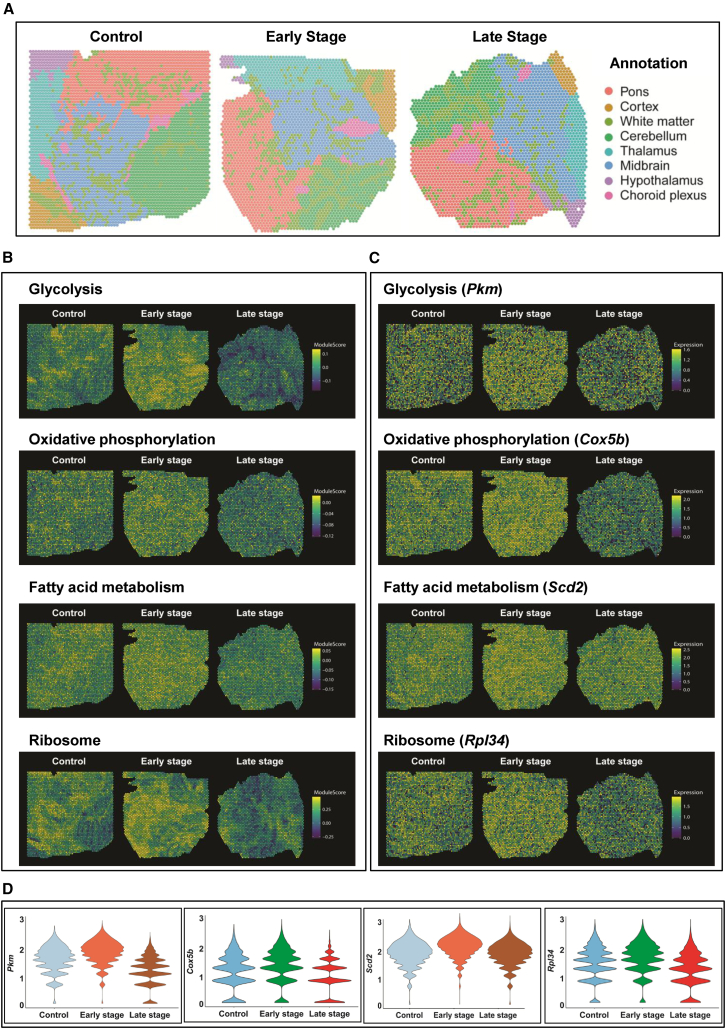
Spatial maps depicting the expression of select HALLMARK gene sets and transcripts in relation to the stage of aSyn pathology in brains of M83^+/+^ mice (A) Representative spatial maps depicting regional annotations within the control, early stage, and late stage M83^+/+^ cohorts using atlas-guided neuroanatomical topography and abundance of regional markers (in A; also see STAR Methods). (B) Representative spatial maps depicting the relative expression of select HALLMARK gene sets (glycolysis, oxidative phosphorylation, fatty acid metabolism, and ribosome/mRNA translation machinery) in cohorts of M83^+/+^ mice. (C and D) Representative spatial maps (in C) and violin plots (in D) depicting the relative expression of select transcriptomics markers within the pathways (shown in B). Abbreviations in 3C-D: *Pkm*, = pyruvate kinase, *Cox5b*, = subunit 5B of cytochrome *c* oxidase, *Scd2*, = stearoyl-CoA desaturase, and *Rpl34*, = a component of large ribosomal subunit.

These gene expression profiles were then used for gene set enrichment (GSE) analyses using HALLMARK and Kyoto Encyclopedia of Genes and Genomes (KEGG) gene sets available on the MSigDB portal (https://bioinf.wehi.edu.au/software/MSigDB/). Gene ontology of global profiles (shown in Figure 2F) revealed that ES was associated with significant upregulation of ATP metabolic/biosynthetic processes (GO:0046034, GO:0072521, GO:0006735, GO:0061621, GO:0006091, GO:0006754, GO:0006090, GO:0030516, GO:0006119); in contrast, LS was associated with downregulation of metabolic processes and cytoplasmic translation (GO:0002181, GO:0006412, GO:0006119, GO:0046034). Moreover, LS was also associated with perturbed regulation of membrane potential, microtubule transport and synaptic vesicle exocytosis (GO:1903829, GO:0032886), neuronal apoptosis (GO:0051402), and chromatin remodeling (GO:0006338, GO:0051402). Key examples reflecting the biphasic expression patterns between the ES and LS are highlighted by spatial maps of select pathways (Figure 4B), or individual genes (spatial plots in Figure 4C and violin plots in Figure 4D). For instance: (i) glycolysis (*Pkm*, encoding a pyruvate kinase isoform, also known as the cytosolic thyroid hormone-binding protein), (ii) oxidative phosphorylation (*Cox5b*, encoding the subunit 5B of cytochrome *c* oxidase in the mitochondrial respiratory complex IV), (iii) fatty acid metabolism (*Scd2*, encoding stearoyl-CoA desaturase, involved in the biosynthesis of fatty acids and mitochondrial beta-oxidation), and (iv) *Rpl34*, which encodes a component of large ribosomal subunit.

**Figure 5:**
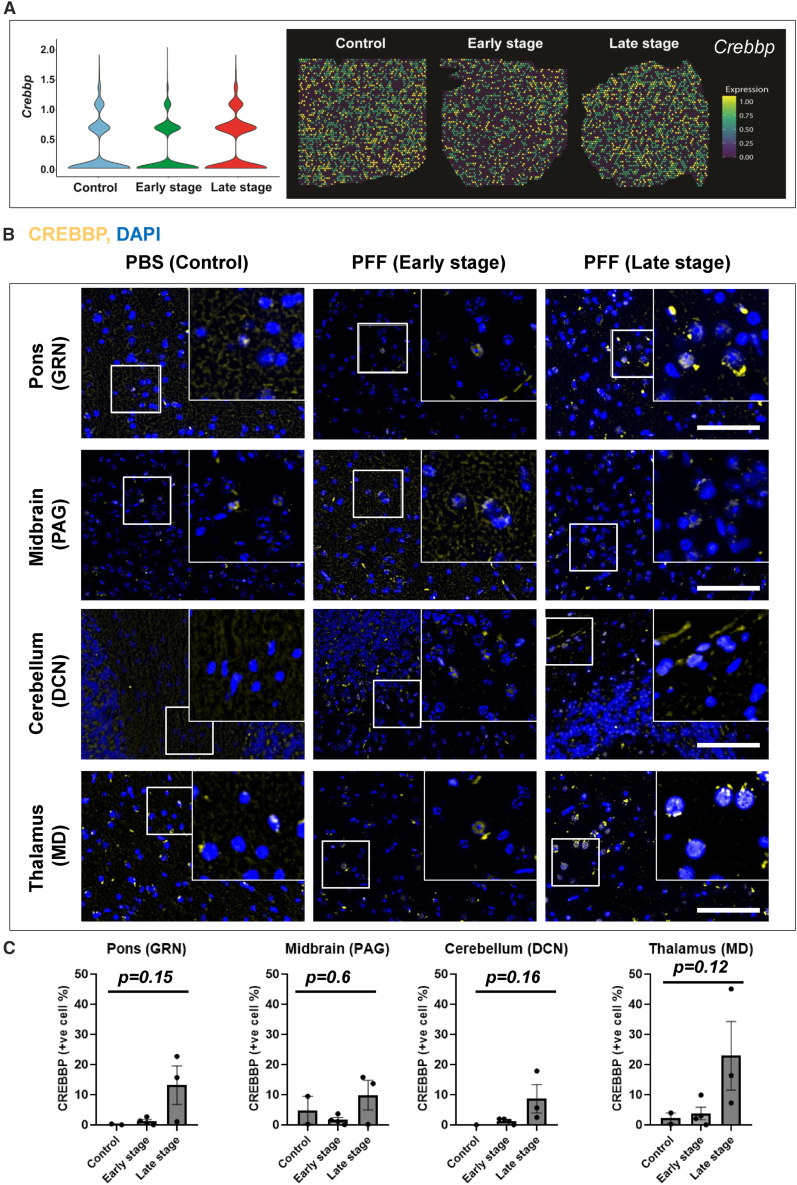
ST maps and immunofluorescence (IF) detection of CREBBP/CBP in brains of M83^+/+^ mice (A) Violin plot and representative spatial maps depicting the relative abundance of *Crebbp* in brains of M83^*+/+*^ mice. (B) Representative (20×) images showing CREBBP/CBP IF in the gigantocellular nuclei (GRN, in pons), periaqueductal gray (PAG, in midbrain), deep cerebellar nuclei (DCN, in cerebellum), and mediodorsal nuclei (MD, in thalamus). The insets show 63× magnified views from the regions. Scale bar, 50 μm. (C) Bar graphs depicting quantification of CREBBP/CBP IF in GRN, PAG, DCN, and DM of the experimental cohorts, as indicated. Error bars depict mean IF intensity ±SD as % of cells in total area (PBS, *n* = 2; DPI-45, *n* = 3; and DPI-75, *n* = 3; see STAR Methods). Statistics in (C): Kruskal-Wallis ANOVA (*p*-values on graphs), Dunn multiple comparisons (non-significant).

In order to decipher how these processes could be linked to perturbations in cellular signaling, we also subjected the ST profiles to cell-cell communication analyses using CellChat.^24^ These analyses revealed distinct ligand-receptor signaling patterns associated with ES and LS (Figures S3A–S3C). For instance, the factors mediating GAS (gaseous signaling molecules), NT (neurotensin), VEGF (vascular endothelial growth factor), and MK (Midkine) signaling were unique to the ES (Figure S3C). In contrast, SLIT (slit guidance ligand) signaling was uniquely associated with the LS. Moreover, PTPR (receptor-like protein tyrosine phosphatase), PACAP (pituitary adenylate cyclase-activating polypeptide), PSAP (prosaposin), and FGF (fibroblast growth factor ) signaling exhibited a progressive decline, while IGF (insulin-like growth factor) signaling exhibited progressive increase during the progression from ES to LS (Figure S3C). Interestingly, some signaling pathways were unique to the cohorts with aSyn pathology (i.e., not enriched in controls) as exemplified by opioid, KIT (tyrosine-protein kinase/stem cell growth factor receptor Kit), GRN (Paragranulin—not to be confused with gigantocellular nuclei, for which we have also used the same abbreviation in Figure 1, Figures 5 and S5), and SPP1 (osteopontin/secreted phosphoprotein 1). The latter data are displayed in Figures S3C and S3F (violin plot and representative spatial maps depicting *Spp1* expression in the ST dataset). Moreover, it is noteworthy that SPP1 (ligand) signaling predominantly originates in pons (one of the early regions affected in this model of aSyn pathology following intramuscular PFF injection),^13^^,^^14^ with receptor interactions involving choroid plexus and hypothalamus (in ES), followed by pons and midbrain (in LS); Figures S3D and S3E.

In addition to these global analyses of the ST data *vis-à-vis* the stage of aSyn pathology, we also subjected the regional ST profiles (Figure S1A and Tables S2–S5) to GSE. These detailed analyses also revealed a biphasic expression of HALLMARK pathways in relation to the stage of aSyn pathology in particular glycolysis, oxidative phosphorylation, lipid/cholesterol homeostasis, and mTORc1 signaling (Figure S1B). Intriguingly, the ES was also associated with augmentation in pathways controlling reactive oxygen species metabolism and DNA repair, which were detected across the gray and white matter annotations (Figure S1B). Moreover, the disease-affected regions in LS (pons, midbrain, and white matter) reflected significant enrichment of the transcripts belonging to pro-inflammatory and tissue remodeling processes (inflammatory response, interferon alpha and gamma, complement, TNF signaling via NF-κB, G2M checkpoint, mitotic spindle, KRAS signaling); Figure S1B. Of note, annotations encompassing regions with minimal aSyn pathology in the model (cortex, thalamus, and hypothalamus) exhibited comparatively lower enrichment of these pathways (Figure S1B).

Lastly, we found a few unique expression patterns in subset of pathways, which were predominantly restricted to distinct ST annotations (Figure S1B). For instance, E2F targets (cerebellum and midbrain), PI3K-AKT-mTOR signaling (cerebellum and cortex), protein secretion (hypothalamus and midbrain), estrogen response (pons and choroid plexus), and hedgehog signaling (thalamus and cortex). Apart from the DGE profiles, a number of individual transcripts exhibited a global upward trend, i.e., upregulated in ES and further increase in LS (e.g., *mt-Nd4l, mt-Atp8, Spp1, Apod, Rbm3, Gabra1, Adcy1*). Conversely, we also noticed a few transcripts that exhibited a continuous downward trend in the global expression (e.g., *Col1a1, Scg2, Lrrc17, Syt1, Syt4, Hsp90b1, Ncam1, Gap43, Rps2, Ndn, Rpl9, Cartpt*). Additional KEGG HALLMARK gene sets/individual gene expression profiles in our ST study can be visualized using the online interface (URL: https://dreamapp.biomed.au.dk/PD_spatial_mouse_DB/; a short User Guide is also included in the Supplementary File S1).

### Curated analyses of patient-derived microarray datasets indicate distinct molecular signatures of aSyn pathology in PD brain

Next, we wanted to investigate if the DGE profiles in the brains of M83^+/+^ mouse model are recapitulated in gene expression studies involving PD patients. For this purpose, we performed curated gene expression analyses of patient-derived microarray datasets using the Gene Expression Omnibus (GEO) repository, as described previously.^25^ The datasets and brain regions (encompassing midbrain and pons/medulla) examined for this purpose include: (1) GSE26927 (SN),^26^ (2) GSE7621 (SN),^27^ (3) GSE20146 (striatum/globus pallidus interna, GPi),^28^ and (4) GSE43490 (SN, dmX and LC).^29^ In these analyses, we prioritized transcripts in the ST data (Tables S2–S5), which were distinctly enriched within the corresponding disease-affected regions in ES and LS (pons, midbrain, and white matter). Altogether, this corresponded to 1,325 unique transcripts (i.e., ES: 142 upregulated, 132 downregulated and LS: 395 upregulated, 656 downregulated), whose expression was then probed across the 4 PD microarray datasets listed above (Table S6).

Table S6. Complete profiles for 1325 select transcripts across PD microarrays

In brief, we discovered several upregulated transcripts from the ST dataset (i.e. mouse model), which exhibited a similar trend in the PD microarray studies (Figure S4A). Among others, increased expression (LogFC≥0.25) of *SPP1* was detected across all microarrays datasets except GSE20146 (GPi); however, this pattern was not consistently significant (based on *p*-value). Notably, few transcripts were consistently upregulated within at least 3 datasets and/or regions, including *KCNJ10, GPR37, CREBBP, NUFIP2*, *and ROCK2* (Figure S4A). In addition, we found quite a few downregulated transcripts from the ST dataset, whose expression was significantly altered in PD microarrays. However, the pattern of change (i.e., upregulation or downregulation) was not consistent, e.g., *NRXN3, ELAVL4, SLC18A2,* GABARAPL1*, ACHE* (Figure S4B). Taken together, these observations point to distinct molecular signatures of aSyn pathology in the nervous system and potentially indicate cellular pathways affected in the disease (further elaborated under discussion).

### Expression of CREBBP protein was increased in the brains of M83^+/+^ mice and in postmortem PD brains

Next, we investigated whether the transcriptional response in the ST data and some of the gene expression profiles in the PD microarray datasets are also reflected in protein expression profiles in relation to the stage of aSyn pathology. We noticed that the majority of transcripts in the ST data that exhibited a consistent pattern of expression in the PD microarray datasets were associated with the late stage of aSyn pathology in M83^+/+^ mice (Figures S4A and S4B). Among the latter, we chose to examine the expression of CREBBP, a 265 kDa histone acetyltransferase, which is ubiquitously expressed in neuronal and glial cells (also known as CBP/KAT3A; not to be confused with CBP/p300, which is structurally and functionally similar to CREBBP). Crucially, the choice of pursuing this factor in subsequent validation analyses was also dictated by the fact that *CREBBP* was significantly upregulated across the PD microarray datasets examined in this study (3 different brain regions except LC/GSE43490, see Figure S3A). At the cellular level, CREBBP binds phosphorylated CREB (cAMP-responsive element binding protein 1) in nucleus and enhances its transcriptional activity,^30^ including c-AMP-responsive gene expression during dopaminergic neurotransmission.^31^

A closer inspection of the ST data indicated a modest but significant increase in *Crebbp* expression in disease-affected regions in the LS cohort vs. ES (i.e., white matter: log2 FC = 0.26, adjusted *p*-value = 3.03^e−24^; Pons: log2 FC = 0.19, adjusted *p*-value = 3.48 E^−10^; Midbrain: log2 FC = 0.19, adjusted *p*-value = 9.96e^−05^ and). This modest increase in *Crebbp* expression was also reflected in data visualization tools, including the violin plots and spatial maps for *Crebbp* (Figure 5A). IF analyses of brain sections also supported a progressive increase in CREBBP protein expression in the LS cohorts compared with the controls or ES cohorts (Figures 5B and 5C). Admittedly, due to the small size of the cohorts, these data did not reach statistical significance (also compare Figure 1C: illustrating aSyn pathology, which also did not reach statistical significance despite abundant *p*-aSyn immunodetection). Intriguingly, the largest effect on CREBBP expression was seen in the mediodorsal (MD) thalamic nuclei (Figure 5C; compare MD with GRN, PAG, and DCN), an area with sparse aSyn pathology in the model (Figure 1C). Altogether, these data suggest that progressive aSyn pathology in the brains of M83^+/+^ mice promotes transcriptional upregulation of *Crebbp* and increased CREBBP protein expression; however, this response is not correlated to the extent/burden of *p*-aSyn (S129) deposition.

For comparison, we performed IF detection of ROCK2 (Rho associated coiled-coil containing protein kinase 2) in the brains of M83^+/+^ mice, since increased expression (neuronal and glial) of ROCK isoforms has been reported in Alzheimer's disease (AD), PD, and atypical parkinsonism.^32^^,^^33^ This pleiotropic serine/threonine kinase is expressed throughout the neuraxis and is the predominant isoform (compared with ROCK1) in the nervous tissue.^34^ In response to activation by Rho GTPases, ROCK2 phosphorylates several cellular targets involved in cytoskeletal organization, cytokinesis, and neurite stability.^34^ Moreover, ROCK2 was also identified as a disease-relevant pathway in association with LS in the rodent model (Figure S3B, *Rock2*), and across 3 different brain regions in 2 PD microarray datasets (Figure S4A, *ROCK2* in GSE7621 and GSE43490). IF analyses indicated substantially enhanced expression and/or localization of ROCK2 in disease-affected regions in the rodent model (Figures S5A–S5C; compare GRN, PAG, and DCN with DM; also see Figure 1C). Taken together, these data (Figures 5 and S5) serve as examples for the validation of candidate markers identified in the ST data and establish their pathological relevance by measuring protein expression in relation to the stage of aSyn pathology.

In this context, while a putative pathologic relevance of SPP1^35^ and ROCK2^32^^,^^33^ to neurodegeneration in PD has been suggested previously, a potential role of aberrant CREBBP expression/regulation in PD remains largely uncharacterized. Related to this, a few examples in the published literature implicate that aSyn aggregation perturbs the cytoplasm to nuclear transport of a related CREB regulator molecule CBP/p300 and its histone acetyltransferase activity.^36^^,^^37^ Therefore, given the novelty and potential functional relevance (regulation of CREB-mediated gene transcription in dopaminergic neurons^31^), we considered that further validation of CREBBP protein expression in the disease-affected regions in PD represents an interesting prospect for further strengthening the scope of our present study.

**Figure 6:**
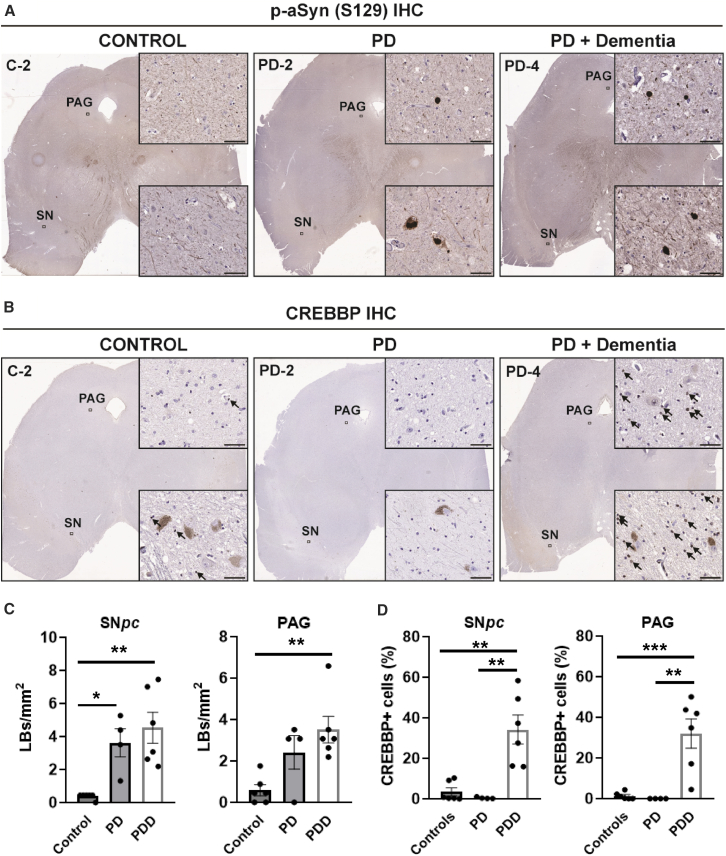
Immunohistochemical (IHC) detection of phosphorylated alpha-synuclein (*p*-aSyn, S129) and CREBBP/CBP in postmortem human brain sections (A and B) Representative images showing IHC detection of *p*-aSyn (S129; in A) and CREBBP/CBP in (B) in postmortem midbrain sections from controls and PD cases (Table S1). Insets show high-magnification images from the substantia nigra pars compacta (SN*pc*) and periaqueductal gray (PAG), reflecting prominent Lewy-related aSyn pathology in PD and visible lack of staining in the controls. Scale bar in insets, 50 μm. The arrows within the inset point to positive instances/cells with CREBBP IHC detection in control and PD midbrain sections. (C) Bar graphs depicting quantification of Lewy body pathology (*p*-aSyn, S129) in the SN and PAG. Graphs display mean ± SEM of Lewy bodies (LBs) per mm.^2^ (D) Bar graphs depicting quantification of CREBBP/CBP IHC in the SN and PAG, expressed as mean % ± SEM of immunopositive cells in the region (see STAR Methods). Data in (C and D) reflect immunopositive counts in *n* = 6 20× views/region/case or control. Statistics in (C and D): One-way ANOVA with Tukey pairwise comparisons (∗p = 0.05; ∗∗p = 0.01; ∗∗∗p = 0.001; only the significant differences are highlighted). Also see Figure S6.

For this purpose, we obtained postmortem midbrain sections from controls (*n* = 4; no clinically known neurological deficit), PD (total *n* = 10 cases comprising PD without dementia, *n* = 4), and clinical diagnosis of PD compounded by dementia, *n* = 6; see Table S1). First, we confirmed the extent of aSyn aggregate pathology in SN and PAG regions by *p*-aSyn (S129) immunohistochemistry (IHC) (Figure 6A). As expected, these analyses revealed significant *p*-aSyn (S129) immunopositive cells in PD midbrain. This was observed in the SN (Figure 6C, showing cells/mm^2^ in controls: Mean = 0.37 ± SEM = 0.17; PD: Mean = 3.62 ± SEM = 0.65, adjusted *p*-value vs. controls = 0.0224, and PD with dementia: Mean = 4.53 ± SEM = 0.61, adjusted *p*-value vs. controls = 0.0019), as well as in the PAG (Figure 6C, showing cells/mm^2^ in controls: Mean 0.58 ± SEM = 0.33; PD: Mean = 2.41 ± SEM = 0.63, adjusted *p*-value vs. controls = 0.1170, and PD with dementia: Mean = 3.51 ± SEM = 0.51, adjusted *p*-value vs. controls = 0.0052).

In serial midbrain sections, we performed IHC analyses for CREBBP expression in control and PD cases (Figure 6B). Remarkably, these analyses revealed significantly increased proportion of CREBBP immunopositive cells in subset of PD cases with dementia compared with the controls, and also compared with PD cases without dementia, in both the SN (Figure 6D showing CREBBP+ cells (%) in controls: Mean = 3.47 ± SEM = 0.89; PD: Mean = 0.44 ± SEM = 0.31, adjusted *p*-value vs. controls = 0.90, and PD with dementia: Mean = 34.14 ± SEM = 0.17, adjusted *p*-value vs. controls = 0.0011 and vs. PD = 0.0012) and the PAG (Figure 6D showing CREBBP+ cells (%) in controls: Mean = 1.28 ± SEM = 0.53; PD: Mean = 0.14 ± SEM = 0.03, adjusted *p*-value vs. controls = 0.98, and PD with dementia: Mean = 32.03 ± SEM = 1.71, adjusted *p*-value vs. controls = 0.0008 and vs. PD = 0.0015). Notably, melanin-containing neurons in the SN exhibited paucity of CREBBP, and the IHC staining was predominantly localized in cells with smaller nuclei and cell size, both in controls and PD cases (Figure 6B, indicated by arrows). A similar staining pattern was also observed in the PAG, such that neurons with large soma were not strongly immunopositive for CREBBP. Further examination of the quantitative IHC data suggested that aSyn pathology (*p*-aSyn, S129) and CREBBP immunopositivity were not strongly correlated, either in the SN (Spearman correlation coefficient = 0.163) or the PAG (Spearman correlation coefficient = 0.022); also see Figures S6A and S6B. Collectively, despite small sample size (for both the animal and human cohorts), these results (Figures 5 and 6) are encouraging findings to further explore the pathological relevance of CREBBP and related molecular pathways in PD.

## Discussion

Circumstantial evidence implicates that the process of aSyn aggregation is a potent stress factor that disrupts neuronal function and cellular homeostasis in the nervous system during the pathogenesis of PD.^1^^,^^38^ Therefore, preventing aSyn aggregation and/or therapeutically modulating cellular pathways of aSyn neurotoxicity represent highly desired pursuits in clinical drug development for PD and related diseases.^39^ Moreover, there are suggestions that pathological aSyn deposition in the nervous system begins several years prior to the onset of motor disability seen in clinical parkinsonism.^1^^,^^2^^,^^5^^,^^9^^,^^17^ Accordingly, the molecular determinants of neuronal resilience and/or vulnerability to incipient aSyn pathology, which potentially contribute to the heterogeneity in clinical presentation of PD subtypes, are increasingly being explored.^40^^,^^41^

Previously, single-nucleus RNA sequencing (RNA-seq) has been applied to midbrain, striatum, and cerebellum of 6-months-old M83 mice, with age-matched non-transgenic controls.^42^ Among the salient findings, the authors reported upregulation of NF-κB signaling, neuroinflammation, peptide metabolic processes, protein synthesis, and protein sorting in trans-Golgi network. In contrast, the majority of downregulated transcripts belonged to pathways involving ion channels, neurotransmitter release, and Rab GTPase signaling. However, no indication of neuropathological analyses, in particular the extent and distribution of aSyn aggregation, was provided.^42^ In the present study, we applied ST to brain sections obtained from a mouse model of extra-nigral synucleinopathy in brainstem.^10^^,^^13^^,^^14^ Our ST data support a mechanistic model (graphical abstract) whereby progressive aSyn pathology triggers a compensatory global transcriptional response affecting metabolic pathways during the early stages. However, with further buildup of aggregated protein(s) a threshold is reached, which heralds an impending energy crisis leading to defective mRNA translation and neurotransmission (Figure 2F). Whether this mechanistic model explains the phenotypic progression from ES to LS in the rodent model remains to be determined experimentally in carefully designed studies. At a glance, our observations regarding the upregulation of metabolic pathways by early aSyn pathology (Figures 2F, 3, and 4) appear counter-intuitive to the prevailing notion, which implicates mitochondrial deficiency and impaired mitochondrial respiration as critical drivers of neuronal loss during the pathogenesis of PD.^43^^,^^44^ This notion is also supported by the fact that direct application of mitochondrial toxins 1-methyl-4-phenyl-1,2,3,6-tetrahydropyridine (MPTP), rotenone, or 6-hydroxydopamine is a highly reproducible method for inducing PD-like dopaminergic cell loss in experimental models.^45^ Moreover, several microarray bulk RNA-seq and single-cell RNA-seq studies also reinforce the etiological relevance of mitochondrial dysfunction to progressive neuropathology in PD (examples in^46^^,^^47^^,^^48^; also see detailed review of related studies including limitations^49^). In this context, our ST data indicate that molecular signatures of mitochondrial deficiency in the brains of M83^+/+^ mice (Figure 2F) represent an advanced stage of aSyn pathology and has implications for neurodegeneration in PD.^43^^,^^44^ Interestingly, recent findings in amyloid-precursor protein knockin mice reflect similar biphasic transcriptomic alterations in mitochondrial metabolic pathways, in relation to progressive neurodegenerative amyloid pathology *in vivo.*^50^

An additional finding that increases the confidence of our ST findings relates to perturbed expression of ribosomal mRNA translation machinery (Figure 2F; downregulated in LS) and corroborates the observations that highlight the defective expression of several initiation and elongation factors in neurodegenerative diseases, including PD (reviewed elsewhere^51^). Moreover, a substantial body of literature purports derangements in ribosomal translation in PD and related neurodegenerative conditions, due to the hyperphosphorylation of eukaryotic translation initiation factor-2 (eIF2α),^52^ eukaryotic elongation factor-2 (eEF2),^53^ AMP-responsive kinase (AMPK),^54^ and mTOR and its signaling targets.^55^ The expected net effect of these biochemical changes in the homeostatic regulation of mRNA translation machinery is a decline in neuronal protein synthesis and activation of the integrated stress response.^51^^,^^55^

Supporting this notion of stressful cellular milieu, our ST data indicate that Spp1 signaling is increased in the brains of M83^*+/+*^ mice during the progression of aSyn pathology (Figure S3). This multifunctional glycoprotein is implicated in immune modulation and acts as a pro-inflammatory cytokine, due to its role in promoting the activation of microglia.^35^ Moreover, the pathogenic relevance of this pathway is also reinforced by similar findings in PD microarray datasets (Figure S4A, *SPP1*) and studies reporting increased osteopontin levels in the cerebrospinal fluid and serum in some cohorts of PD patients.^35^ Related to this, a crucial effector mechanism in tissue damage associated with neurodegenerative aggregate pathology involves the activation of ROCK2 (a serine/threonine kinase). Growth inhibitory signals in neuronal milieu are potent activators of the RhoA/ROCK2 pathway, which in turn leads to growth cone collapse and axonal degeneration (for instance, through phosphorylation of LIM domain kinase).^56^ Increased ROCK2 expression and/or markers of RhoA/ROCK2 activity are linked to several neurodegenerative conditions, including PD and atypical parkinsonism.^32^^,^^33^ Not surprisingly, our ST data also corroborate the pathological relevance of ROCK2 in brains of M83^+/+^ mice, as demonstrated by increased *Rock2* transcript enrichment (Figure 3B), and IF detection of ROCK2 protein in association with LS aSyn pathology (Figures S5B and S5C).

Among the signaling pathways, the ST data indicate that IGF signaling exhibited progressive increase during the progression from ES to LS (Figure S3C). IGF-1 signaling plays a crucial role in brain development and synaptogenesis, and stimulates neuroprotective repair processes following CNS injury.^57^ Moreover, IGF-1 and related family members help regulate blood glucose by increasing insulin sensitivity and decreasing hepatic gluconeogenesis. Hence, perturbations in IGF-signaling have been implicated in pro-inflammatory states,^58^ type 2 diabetes mellitus (T2DM) and metabolic syndrome.^59^ In this context, the prevalence of clinically diagnosed T2DM in PD has been reported to be as high as 8–30%, and systemic insulin resistance was found in 50–80% in cohorts of PD patients.^60^^,^^61^ Some studies indicate that T2DM comorbidity is associated with decline in dopamine transport in the striatum and worsens the progression of motor disability in PD.^62^^,^^63^^,^^64^ A proximate link between aSyn and blood glucose regulation has also been suggested by some studies in which peripheral (intraperitoneal) delivery of monomeric aSyn increased glucose tolerance in wild-type mice and modulated glucose-stimulated insulin secretion by pancreatic beta cells *in vitro*, putatively through increased membrane retention of glucose transporter GLUT4.^65^

While the above findings highlight defective homeostatic regulation of energy balance and/or neuroinflammation in response to progressive aSyn pathology, it is challenging to pinpoint their direct relevance to etiology of specific PD symptoms. It is increasingly being recognized that non-motor symptoms (sleep disturbances, pain, olfaction, and autonomic dysfunction) adversely affect the quality of life in PD^1^^,^^2^ and could arise due to disturbances in the circadian regulation of brain metabolism and neuronal signaling.^66^ The molecular and genetic evidence to support this notion in clinical studies and animal models is only recently beginning to emerge (reviewed elsewhere^67^^,^^68^). In this context, CREBBP/CBP has been suggested to act as a direct activator of circadian complex CLOCK/BMAL1 in the nucleus, which then binds to enhancer elements in promoter regions of core clock gene Period 1 (*PER1*).^69^ Moreover, moderate (∼50%) increase in the expression of CREBBP in *Drosophila* disturbs their rhythmic locomotor activity in darkness without directly engaging the circadian clock machinery.^70^ It is noteworthy that altered sleep-wake cycles and disturbances in circadian rhythm have also been reported in some rodent models of synucleinopathy.^71^^,^^72^^,^^73^ Moreover, we have recently reported that aSyn aggregate pathology in the GRN of PFF aSyn-injected M83^+/−^ mice led to progressive decline in nocturnal activity in home cage long before features of movement disability were manifest.^74^ Therefore, with reasonable caution, our findings related to the perturbed expression CREBBP/CBP protein in brains of M83^+/+^ mice (Figure 5) and cases with advanced PD + dementia (Figure 6) are potentially relevant and worth further investigation.

In a broader context, it remains to be determined if the spatiotemporal features of transcriptomic changes identified in the model used in this study (murine PFF aSyn injected in transgenic M83^+/+^ mice) are recapitulated in different paradigm(s) of aSyn aggregation. In particular, there is burgeoning evidence that aSyn aggregates exist in distinct conformational states, also termed strains, which in turn are thought to underlie heterogeneity in the clinical presentation of PD and related synucleinopathies.^5^^,^^6^ In this regard, recent research in animal models suggests that depending on the source, fibrillization conditions, and route of entry in the CNS, distinct aSyn strains can induce unique features of cellular pathology. For instance, some aggregate strains can be low in amyloidogenic dye binding, a standard criteria for validating the fibrillar nature of aSyn, but are potent inducers of *de novo* aSyn aggregation in neuronal cultures and *in vivo.*^75^ In related studies, intracerebral inoculation of different aSyn strains, or brain homogenates obtained from moribund M83^+/+^ mice or Multiple system atrophy patients, was found to differentially affect the distribution and burden of aggregated aSyn in brains of M83^+/+^ mice, as well as dictate distinct pattern of movement disability.^76^^,^^77^ Hence, determining the strain-driven cellular effects of aSyn is an attractive prospect for future investigations and holds promise for the discovery of potential biomarkers for PD subtypes.

In conclusion, we describe the molecular signatures associated with progression of PD-like aSyn pathology in the nervous system of a rodent model. Our findings support a mechanistic paradigm whereby the neurotoxic effects of pathological aSyn deposition are potentially thwarted/masked by metabolic compensation conferred by elevated energy flux through mitochondrial pathways. With the progression of the disease, our data support the notion that the failure of mitochondrial metabolism heralds the onset of transcriptional reprogramming potentially linked to neurodegeneration in PD.

### Limitations of the study

The most significant limitation of the study is the small sample size for the animal study (controls, *n* = 2; ES PFF, *n* = 3; and LS PFF, *n* = 3) and limited histological analyses (Figure 6) in human midbrain sections (controls, *n* = 6; PD without dementia, *n* = 4; and PD with dementia, *n* = 6). This limitation precludes the generalizability of the findings, especially for determining the influence of sex and age on the results of the study. Second, the technological platform (Visium V1) lacks single-cell resolution; hence cell-type-specific effects of aSyn pathology in the ST datasets are not directly inferred. Third, due to the observational nature of the study, we cannot conclusively establish whether the changes in gene expression in rodent brain (Figures 3 and 4) or PD microarrays (Figure S4) are consequences of aSyn aggregation and/or represent ancillary mechanisms in disease pathogenesis. Fourth, despite the reproducible nature of PFF-based models including the transgenic M83 line for modeling synucleinopathies, they do not recapitulate the full spectrum of human PD. Notably, this pertains to the lack of neurodegeneration in the SN and paucity of aSyn aggregation in midbrain dopaminergic neurons, as well as limited scope for studying the effects of co-pathologies associated with subtypes of PD (e.g., Tau). Lastly, the significance of increased CREBBP expression in PFF cohorts (Figure 5) and PD cases with dementia (Figure 6) remains unknown. Further systematic studies are warranted to establish whether this pathway is preferentially activated in synucleinopathies or represents a general neurodegenerative process associated with dementia.

## Resource availability

### Lead contact

Requests for further information and resources should be directed to and will be fulfilled by the lead contact, Asad Jan (ajan@aias.au.dk).

### Materials availability

All reagents and materials generated or used in this study are commercially available. Detailed information regarding the suppliers and catalog numbers is provided in the key resources table.

### Data and code availability

• Data: All of the data generated and analyzed during this study are included in the main manuscript and the associated supplementary files. The processed and raw RNA-seq data generated during this study are publicly accessible through the NCBI GEO repository (accession number: GSE274605).
• Code: No new code was generated in the study.
• Other: For the visualization and interactive exploration of the ST data, we have created a dedicated online interface (URL: https://dreamapp.biomed.au.dk/PD_spatial_mouse_DB), and a short user guide is included in the supplemental PDF (Data S1). This interactive platform has been developed and customized using the ShinyCell package. Any additional information required to reanalyze the data reported in this paper is available from the lead contact upon request.

## Acknowledgments

This work was supported by funding to A.J. in the form of a Marie Skłodowska Curie Fellowship from European Union’s Horizon 2020 Research and Innovation Programme (MSCA-IF-2017, grant #786433). Funding for P.H.J. was from the 10.13039/501100003554Lundbeck Foundation (grants R223-2015-4222 for DANDRITE, Nordic-EMBL Partnership for Molecular Medicine, AU). The authors would like to thank Trine Werenberg Mikkelsen for the help and assistance with the preparation for mouse brain sections for the ST study.

## Author contributions

L.L., A.D., and A.J. designed research; L.L., N.M.J., S.A.F., A.D., and A.J. performed research; L.L. performed bioinformatics analyses and designed the online visualization platform; N.M.J., S.A.F., F.Y., and A.J. analyzed data; I.R.M. provided human postmortem tissue and contributed to discussion; P.H.S., M.R.-R., P.H.J., and J.R.N. contributed with infrastructural and personnel support; L.L., N.M.J., and A.J. wrote the manuscript. All the authors read and approved the manuscript.

## Declaration of interests

The authors declare no competing interests.

## STAR★Methods

### Key resources table

**Table:** 

REAGENT or RESOURCE	SOURCE	IDENTIFIER
**Antibodies**		

Anti-phosphorylated alpha-synuclein (S129), EP1536Y	Abcam	Cat#ab51253; RRID: AB_869973
Anti-CREBBP	Abcam	Cat#ab253202; RRID: AB_3676065
Anti-ROCK2	LSBio	Cat#LS-C3334228; RRID: AB_3738383
Alexa-Fluor488 fluorophore conjugated secondary antibody	Thermo Fisher	Cat#A-11008; RRID: AB_143165

**Bacterial and virus strains**		

BL21(DE3) competent cells (recombinant mouse alpha-synuclein expression)	PHJ, co-author	Not applicable

**Chemicals, peptides, and recombinant proteins**		

Recombinant (wild type) mouse αSyn	PHJ, co-author	Not applicable
Phosphate-buffered saline	Thermo Fisher	Cat#10010023
Heparin	Sigma	Cat#H3149
Methanol	Sigma	Cat#34860
Tissue-Tek OCT	Sakura	Cat#4583
Hematoxylin	Vector Labs	Cat#H-3401
Paraformaldehyde, 4%	Electron Microscopy Sciences	Cat#15700

**Critical commercial assays**		

Visium Spatial Gene Expression Reagent Kit	10× Genomics	Cat#1000184
Visium Spatial Gene Expression Slide Kit	10× Genomics	Cat#1000185
Library Construction Kit	10× Genomics	Cat#1000190
Dual Index Kit TT Set A	10× Genomics	Cat#1000215
Pierce™ BCA Protein Assay	Thermo Fisher	Cat#23250
Alkaline phosphatase conjugated streptavidin-biotin ABC kit	Vector Labs	Cat#AK-5000

**Deposited data**		

Microarray dataset, PD and controls	NCBI Gene Expression Omnibus Repository	GSE26927
Microarray dataset, PD and controls	NCBI Gene Expression Omnibus Repository	GSE7621
Microarray dataset, PD and controls	NCBI Gene Expression Omnibus Repository	GSE20146
Microarray dataset, PD and controls	NCBI Gene Expression Omnibus Repository	GSE43490
ST dataset, M83 mice	NCBI Gene Expression Omnibus Repository	GSE274605

**Experimental models: Organisms/strains**		

B6;C3-Tg(*Prnp-SNCA∗A53T*)83Vle/J	Jackson Laboratories	Cat#004479

**Recombinant DNA**		

Plasmid DNA, murine full length alpha-synuclein	PHJ, co-author	Not applicable

**Software and algorithms**		

Qupath (v. 0.5.1)	https://github.com/qupath/qupath/releases	https://github.com/qupath/qupath/releases
spaceranger-1.3.0	10× Genomics	https://www.10xgenomics.com/support/software/space-ranger/latest
R (v4.2.3)	The R Foundation	https://www.r-project.org/
ggplot2 (v3.4.4)	https://ggplot2.tidyverse.org/	https://ggplot2.tidyverse.org/
Seurat(v4.3.0)	https://satijalab.org/seurat/	https://satijalab.org/seurat/
msigdbr(v7.5.1)	https://github.com/igordot/msigdbr	https://github.com/igordot/msigdbr
GSVA(v1.46.0)	https://www.bioconductor.org/packages/release/bioc/html/GSVA.html	https://www.bioconductor.org/packages/release/bioc/html/GSVA.html
UpsetR(v1.4.0	https://github.com/hms-dbmi/UpSetR	https://github.com/hms-dbmi/UpSetR
GraphPad Prism v8	GraphPad	https://www.graphpad.com/
ST database, M83 mice	LL, first author	https://dreamapp.biomed.au.dk/PD_spatial_mouse_DB

### Experimental model and study participant details

#### Human participants

The study does not involve human participants except anonymized postmortem human tissue brain section samples provided by IRM (co-author), as approved by the University of British Columbia Ethics Committee. Table S1 contains the relevant demographic profiles, including age and sex, of the controls (n=6) and PD cases (n=10) examined by IHC stainings.

#### Animal studies

Transgenic M83 mice [B6;C3-Tg(*Prnp-SNCA∗A53T*)83Vle/J]^10^ were housed at the Skou animal facility at Aarhus University in accordance with Danish regulations and the European Communities Council Directive for laboratory animals (Danish Animal Experiments Inspectorate Committee/Dyreforsøgstilsynet license # 2017-15-0201-01203 issued to PHJ, co-author). The animals were housed under a 12 hours light/dark cycle and fed with regular chow diet *ad libitum*. Adult homozygous M83^+/+^ mice (12-14 weeks of age) were bilaterally inoculated with a single injection (5 μl) of recombinant mouse aSyn PFF (1 μg/μL; n=6) into the hindlimb biceps femoris, using a 10-μL Hamilton syringe with a 25-gauge needle as described in the foundational study.^13^ Age-matched controls were injected with PBS (5 μl, bilaterally; n=2). The experiments included both male and female mice and animals were randomly allocated to the PBS or PFF cohorts.

#### Collection of brains and preparing tissue sections

Mice were transcardially perfused with ice-cold PBS supplemented with 10 U/ml Heparin (Sigma, #H3149). Brains were removed from the skull and immediately transferred on ice-cold PBS. Then, the brains were embedded in Tissue-Tek OCT (Sakura, #4583), immediately frozen by liquid nitrogen bath and kept at -80°C until cryosectioning. For ST and related analyses in the present study, the OCT embedded hemisphere was initially trimmed from the midline, covering ∼50 μm, to expose the paramedian nuclei (GRN and PAG; see note on Mouse Neuroanatomical Topography below).^22^ Then, five 10 μm thick sagittal serial sections were obtained, with the cryostat chamber maintained at -20°C. The outer four sections, placed on the Superfrost Plus slides (VWR, #631-0108), were stored -80°C and reserved for the immunofluorescence (IF) analyses. The central section, used for ST, was placed within the capture area (6.5 x 6.5 mm) on the Visium Spatial Gene Expression slides (10× Genomics, #PN-1000185), covering brain regions of interest (paramedian pons and midbrain, cerebellum, thalamus, hypothalamus and cortex).^13^^,^^15^^,^^16^

### Method details

#### Generation of mouse aSyn fibrils

Mouse aSyn fibrils (PFF) were prepared and characterized *in vitro*, essentially as described.^12^^,^^13^ Briefly, full length recombinant (wild type) mouse αSyn was expressed in BL21(DE3) competent cells and purified using reverse phase chromatography. Then, purified recombinant monomeric (ie., non-aggregated) α-Syn (10 mg/ml) was incubated at 37°C in phosphate-buffered saline (PBS, pH 7.4) with continuous shaking at 1050 rpm in a tabletop microtubes shaker (Eppendorf). The PFF were collected by centrifugation (15,600g at 25°C for 30 min), and then re-suspended in PBS. Protein concentration was determined by the BCA assay (Pierce) and a stock solution consisting of 2 mg/mL protein was prepared (in PBS). Subsequently, PFF were sonicated for 20 minutes using a Branson 250 Sonifier at 30% intensity, and then aliquoted and frozen at −80°C until further use. The purity of these fibrillar preparations, their biophysical characterization and biological activity is described elsewhere.^12^^,^^13^

#### Spatial transcriptomics

##### Spatial library preparation

Spatial bar-coded ST library from fresh frozen brain sections was prepared using Visium Spatial Gene Expression Slide & Reagent Kit (10× Genomics, #PN-1000184), according to the manufacturer’s provided instructions (CG000240 Rev C). The sections were fixed in pre-chilled (-20°C) methanol (Sigma, #34860) for 10 min at -20°C. Sections were counterstained with hematoxylin (Vector Labs, #H-3401) for 5 min at room temperature (RT) and whole slide digital scans were obtained using the Olympus VS120 upright microscope in the brightfield mode (20× magnification). Samples were permeabilized for 18 min, based on the optimal time determined in the tissue optimization procedure (10× Genomics, #1000193 & Cat#CG000238 Rev D). Spatial cDNA libraries were prepared according to the manufacturer’s guidelines for the procedure (CG000240 Rev C: reverse transcription, second strand synthesis, cDNA amplification and adapter ligation). The resulting libraries were pooled and sequenced on an MGI G400 sequencer, followed by de-multiplexing using the library index sequences.

##### Sequencing data alignment and normalization

Sequencing data in FASTQ format were aligned to the reference genome (refdata-gex-mm10-2020-A) using Space Ranger software (10× Genomics, version 1.3.0). Briefly, spots under tissues were selected in LoupeBrowser, json files were then exported and used for alignment with Space Ranger. The Seurat package was utilized for data normalization and annotation of each cryosection. Raw count inputs were normalized using the SCTransform normalization method. Following normalization, principal component analysis (PCA) was performed for linear dimension reduction, selecting the first 30 principal components (PCs) to create a shared nearest neighbor (SNN) network. The same PCs were employed for visualization using Uniform Manifold Approximation and Projection (UMAP) and t-Distributed Stochastic Neighbor Embedding (t-SNE) algorithms.

##### Spatial annotation

Clusters within each cryosection were annotated by calculating cluster-specific markers using the “FindAllMarkers” function. This determined log fold change, percentage of expression within and outside the target cluster, and p-values for the Wilcoxon rank-sum test. A log2 fold change (FC) threshold of ≥±0.25 and an adjusted p-value ≤0.05 were considered significant. By comparing identified marker genes with canonical region marker genes, each cluster was assigned an ST regional annotation.

##### Merging data and unsupervised clustering

Individual Seurat objects for each cryosection were combined into a single, integrated dataset. Unsupervised clustering was conducted, involving PCA to identify major axes of variation within the data. The first 20 PCs were selected for further analysis, generating an SNN network to reveal the data’s underlying structure. These 20 PCs were used for visualization, applying dimensionality reduction techniques such as UMAP and t-SNE to represent and interpret high-dimensional data more comprehensibly.

##### Correlation analyses

To assess relationships between different regions within each region/cryosection, the average gene expression for all genes in each region was calculated. Pair-wise correlation coefficients between all pairs of regions were computed based on their average gene expression profiles using the 'cor' function from the 'stats' package. A Euclidean distance-based dissimilarity matrix was constructed from the resulting correlation matrix. Hierarchical clustering was then performed on the dissimilarity matrix using the complete linkage method. This approach identified patterns and relationships between distinct regions across cryosections based on their gene expression similarities, providing insights into the underlying biological processes and molecular signatures.

##### Pair-wise comparison of differential gene expression

Differentially expressed genes between two groups were identified using the 'FindMarkers' function in the Seurat package. This function calculates the log2 FC in gene expression, the percentage of expression within and outside the target cluster, and p-values using the likelihood-ratio test. DEGs were considered significant if they met the following criteria: a log2 FC threshold of ≥±0.25 and an adjusted p-value of ≤0.05.

##### Gene set enrichment (GSE) analyses

To investigate the functional relevance of gene expression patterns in our dataset, we employed two distinct strategies for gene set analysis. The gene sets were collected from the Molecular Signatures Database (msigdbr version 7.5.1; http://bioinf.wehi.edu.au/software/MSigDB/) using the msigdbr package, enabling us to identify enriched pathways and cellular processes across different regions and time points.

The first strategy involved performing Gene Set Variation Analysis (GSVA) on the average expression for each cluster. The average expression was calculated for each region in different cryosections. GSVA was conducted using the GSVA R-package (version 1.46.0), which converts the gene-by-cluster matrix into a gene-set-by-cluster matrix, providing a pathway-centric perspective on the data.

The second strategy employed the 'AddModuleScore' function from the Seurat package to calculate module scores for individual gene sets. This approach enabled the computation of module scores for each spot in the dataset, offering a more granular view of gene set activity across the spatial transcriptomic landscape. Together, these complementary strategies allowed for a comprehensive understanding of the biological processes and molecular pathways underlying the observed gene expression patterns in the ST dataset.

##### Data visualization

A range of visualization techniques were employed to effectively represent the ST data. We generated UMAP and spatial plots, violin plots, and gene expression dot plots using the 'DimPlot', 'SpatialDimPlot', 'SpatialPlot', 'VlnPlot', and 'DotPlot' functions within the Seurat package. Additionally, heatmaps were created utilizing the ‘heatmap’ function in the stats package (version 4.0.3). Genes responded in different regions at different time points were analyzed and visualized with UpsetR package. ST data were also integrated and visualized as KEGG pathway maps using Pathview R package.^78^ For the visualization and interactive exploration of the ST data, we have created a dedicated online interface (URL: https://dreamapp.biomed.au.dk/PD_spatial_mouse_DB, and a short User Guide is provided in the Supplementary Information (Document S1).

#### Histological analyses

##### Immunofluorescence (IF) analyses of mouse brains

IF was performed on 10 μm thick fresh frozen sections, after drying (5 min, at 37°C in a non-humid oven) and gentle fixation with ice-cold 4% Paraformaldehyde (Electron Microscopy Sciences, #15700; 15 min, at 4°C). Nonspecific binding was blocked by incubating the sections in 5% normal donkey serum in Tris-buffered saline (1 hour, RT). Then, the sections were incubated (overnight, at 4°C) with the following primary antibodies (in PBS containing 0.3% Triton-X and 0.5% bovine serum albumin- BSA): phospho-S129 aSyn (rabbit mAb EP1536Y, Abcam, #ab51253- dilution: 1:1000), Creb-binding protein (CREBBP/CBP; rabbit mAb EPR23418-23 Abcam, #ab253202- dilution: 1:500) and Rho Associated Coiled-Coil Containing Protein Kinase 2 (ROCK2, rabbit pAb, LSBio#LS-C3334228- dilution: 1:250). IF detection was performed by Alexa-Fluor488 fluorophore conjugated secondary antibody (Thermo Fisher #A-11008- dilution: 1:1000).

##### Mouse neuroanatomical topography

Panoramic images from the digital whole slide scans were mapped onto Mouse Brain Atlas (Paxinos and Franklin's The Mouse Brain in Stereotaxic Coordinates, 4^th^ Edition).^22^ Information about neuroanatomical tracts and nuclei was primarily derived from The Mouse Nervous System (1^st^ Edition).^23^

##### Immunohistochemistry (IHC) analyses of human tissue

IHC analyses on post-mortem control and PD midbrain sections were performed by using p-aSyn (p-S129) antibody (rabbit mAb EP1536Y Abcam, #ab51253- dilution: 1:1000) and anti-CREBBP (rabbit mAb EPR23418-23 Abcam, #ab253202- dilution: 1:500) essentially as described.^53^ Five μm formalin-fixed paraffin embedded sections were kindly provided by IRM (co-author), after the study approval by the University of British Columbia Ethics committee (Table S1). IHC on brain sections was performed after deparaffinization and antigen retrieval. For destaining/bleaching of neuromelanin in the substantia nigra (in p-aSyn S129 IHC studies), a slightly modified IHC protocol was used.^13^^,^^53^ Briefly, slides with mounted sections were incubated in a 60°C degrees oven for 30 minutes and then were transferred into ambient distilled water. Then, the slides were placed in 0.25% potassium permanganate solution for 5 minutes. Subsequently, the slides were rinsed with distilled water. This was followed by incubation in 5% oxalic acid until section became clear. A final rinse in distilled water was performed before proceeding with the routine IHC staining on serial sections as described below. Tissue sections were incubated with the primary antibodies and immunodetection was performed using the alkaline phosphatase conjugated streptavidin-biotin ABC kit (Vector Labs, #AK-5000). Sections were counterstained with hematoxylin (Vector Labs, #H-3401).

##### Image analyses-mouse brain sections

High resolution IF views of the mouse brain sections were obtained using Olympus VS120 digital slide scanner equipped for fluorescence single-band emitters for Hoechst, FITC, Cy3 and Cy5 (AU, Denmark). Slide scans were imported into Qupath (v. 0.5.1)^79^ and entire tissue regions were outlined based on thresholding for the nuclear DAPI staining. Regions of interest (ROI) consisting of GRN (pons), PAG (midbrain), deep cerebellar nuclei (DCN) and mediodorsal (MD) thalamic nuclei were manually outlined in each section, using the Mouse Brain Atlas (Paxinos and Franklin's The Mouse Brain in Stereotaxic Coordinates, 4^th^ Edition)^22^ as a guide. ROI were segmented using the Qupath cell detection plugin on the DAPI channel. Phospho-aSyn (S129) threshold was computed as 10 x median IF intensity in the entire tissue region. This threshold was applied in a Pixel Classification thresholder on the detection channel, using full image resolution and no prefiltering/smoothing. The thresholder was then used to compute p-aSyn (S129)-positive area as percentage (%) of the total ROI area.

For analyzing the IF staining for CREBBP, automated thresholding was performed on the detection channel (script for graphical user interface available at https://github.com/iviecomarti/GUI_AutoTH_QuPath/blob/main/gui/gui_AutoTH_QuPath.groovy). Thresholding was performed on the entire tissue region using the “Moments” method to get a threshold value as output. Subsequently, this threshold was multiplied by 1.2 and put into a Single Measurements Classifier on the CREBBP channel to compute CREBBP-positive cells, defined as having a mean intensity in the nucleus above the set threshold. Results were plotted as CREBBP-positive cells in % of total cells in the ROI. For the analysis of ROCK2 IF staining, median ROCK2 intensity was computed in each ROI and multiplied by 1.5 to get the threshold. This threshold was put into a Single Measurements Classifier on the detections channel to compute ROKC2-positive cells, defined as having a mean intensity in the cytoplasm above the set threshold. Results were graphed as ROCK2-positive cells in % of total cells in the ROI.

##### Image analyses-human brain sections

Anonymized whole slide digital panoramic images of the tissue sections for IHC analyses were acquired using a Leica Aperio slide scanner (UBC, Canada). Slide scans from PD cases and non-neurodegenerative controls were imported into Qupath (v. 0.5.1)^79^, and ROIs of approx. 720 x 500 μm were defined for the SN and PAG in both hemispheres (one in each hemisphere). All analysis was performed blinded to the diagnosis of the cases and CREBBP staining was analyzed before assessing the p-aSyn (S129) stained sections. For the analyses of CREBBP IHC staining, large blood vessels and neuromelanin was first excluded from the ROIs. Then, stain vectors were estimated using the auto function in Qupath. Positive cell detection was run on the ROIs with nuclei detected by hematoxylin stain and positive cells defined by a mean optical density of nuclear DAB staining above 0.2. Results were graphed as CREBBP-positive cells in % of the total cells each ROI. For the p-aSyn (S129) IHC analyses, LBs were manually counted in each ROI and normalized to the total ROI area to obtain the number of LBs per mm2 of the tissue.^53^

### Quantification and statistical analyses

The immunostaining/immunofluorescence data were statistically analyzed in GraphPad Prism software version 8, and the final graphs were prepared in GraphPad or Microsoft Excel. Statistical significance in datasets was calculated and indicated in the relevant figure legends. P-values were set at: ∗p<0.05, ∗∗p<0.01, ∗∗∗p<0.001, ∗∗∗∗p<0.0001.

## Supplemental information

Document S1. Figures S1–S6, Table S1, Data S1

Table S2. Upregulated transcripts, Early stage vs. Controls

Table S3. Downregulated transcripts, Early stage vs. Controls

Table S4. Upregulated transcripts, Late stage vs. Early stage

Table S5. Downregulated transcripts, Late stage vs. Early stage
