# Supplementary material for: Spatial transcriptomics reveals the molecular signatures of prodromal and advanced α-synucleinopathy
Source: iScience. 2026 Jan 29;29(3):114845. doi: 10.1016/j.isci.2026.114845 (PMC12927100; doi:10.1016/j.isci.2026.114845)
Supplement: Document S1. Figures S1–S6, Table S1, Data S1 [file mmc1.pdf]

iScience, Volume *29*

Supplemental information

Spatial transcriptomics reveals the molecular signatures of prodromal and advanced α-synucleinopathy

Lin Lin, Nanna M. Jensen, Alberto Delaidelli, Sara A. Ferreira, Fatemeh Yarmahmoudi, Poul H. Sorensen, Marina Romero-Ramos, Poul H. Jensen, Ian R. Mackenzie, Jens R. Nyengaard, and Asad Jan

Table S1. Demographics of PD cases and controls used in the study							
**Case/Controls**	**age**	**sex**	**Clin Dx**	**LBD**	**Braak**	**SP**	**other**
PD-1	71	M	PD	limbic	II	None	LATE-NC
PD-2	69	M	PD	diffuse	0	none	
PD-3	87	M	PD + dementia	diffuse	IV	frequent	
PD-4	74	M	PD + dementia	diffuse	III	moderate	
PD-5	67	M	PD	limbic	II	none	acute anoxic-ischemia
PD-6	74	M	PD	diffuse	I	none	
PD-7	74	M	PD + dementia	diffuse	III	none	LATE-NC
PD-8	81	F	PD + dementia	diffuse	III	none	
PD-9	69	M	PD + dementia	diffuse	III	moderate	
PD-10	81	M	PD + dementia	diffuse	II	sparse	
C-1	78	M	CVD	none	III	rare	CVD
C-2	93	F	CVD	none	III	rare	CVD
C-3	76	F	CVD				
C-4	50	M	CVD				
C-5	76	M	CVD				
C-6	71	F	Lymphoma				

Abbreviations: C= Control; CVD: chronic cerebrovascular disease; LBD = Lewy body disease/Pathology; PD = Parkinson disease
Notes on pathology:
LBD stage: brainstem (brainstem only), limbic (brainstem + limbic), diffuse (brainstem + limbic + neocortex)
Braak: stage 0 – VI
Senile Plaques: CERAD none, rare, moderate, frequent
LATE-NC: TDP-43 pathology in limbic structures
C-3: *normal population of pigmented neurons with small number of incidental LB.

SUPPLEMENTARY FIGURE LEGENDS

S1. Number of differentially expressed transcripts across annotations and heatmap depicting the expression of HALLMARK gene sets in relation to the stage of aSyn pathology in brains of M83$^{+/+}$ mice. (A) Pie charts indicating the number of differentially expressed gene transcripts across the 8 ST annotations using the cut-off criteria: Log2FC±0.25 and adjusted p≤0.05. **(B)** Heatmap depicting the relative expression of HALLMARKS gene sets in pair-wise comparisons involving Early stage (E) vs. Controls (C) and Late stage (L) vs. Early stage (E) in experimental cohorts of M83$^{+/+}$ mice. Abbreviations in S1B: Crb (cerebellum), Ch. plx (choroid plexus), Ctx (cerebral cortex), hyp. th. (hypothalamus), Mb (midbrain), Thal. (thalamus) and wh. mat. (white matter). Also see Fig. 2F.

S2. Heatmaps depicting the expression of select transcripts (as examples of unique transcriptomic changes) within the ST annotations in relation to the stage of aSyn pathology in brains of M83$^{+/+}$ mice. (A-D) Gene expression profiles indicated by the Log2FC values which were upregulated at early stage (in A), upregulated at late stage (in B), downregulated at early stage (in C) and downregulated at late stage (in D) with adjusted p-value ≤0.05. Also see Fig.4 for the expression of top 50 transcripts across the ST annotations.

S3. CellChat profiles of the differentially expressed transcripts in relation to the stage of aSyn pathology in brains of M83$^{+/+}$ mice. (A-B) Depiction of regionally inferred (between ST annotations) number of cell-cell interactions and their strength in relation to the stage of aSyn pathology in brains of M83$^{+/+}$ mice. **(C)** Bar graph depicting the weighted signal strength (information flow) in distinct signaling pathways in relation to the stage of aSyn pathology in brains of M83$^{+/+}$ mice. **(D-E)** Heatmaps depicting hierarchical clustering of signaling pathways for outgoing (ligand; in D) and incoming (receptor, in E) interactions. The heatmap reflects the relative strength of each signaling pathway with strong interactions (dark green) to weak interactions (white) represented within individual tiles, while the colored bar plot (on top) reflects

the sum of weighted signal strength (on a scale of 0-2 arbitrary units) across signaling pathways. Pathways in C-E: GAS, gaseous signaling molecules; NT, Neurotensin; VEGF, Vascular endothelial growth factor; MK, Midkine; PTPR, Receptor-like protein tyrosine phosphatise; PACAP, Pituitary adenylate cyclase-activating polypeptide; PSAP, Prosaposin; FGF, Fibroblast Growth Factor; PTN, Pleiotrophin; SLITRK, Leucine-rich repeat SLIT and NTRK-like family; NT, Neurotrophin; IGF, Insulin-like growth factor; SLIT, Slit guidance ligand; KIT, Tyrosine-protein kinase/stem cell growth factor receptor Kit; GRN, Paragranulin ad SPP1, secreted phosphoprotein 1/Osteopontin. **(F)** Violin plot and representative spatial maps depicting the relative abundance of *Spp1* in relation to the stage of aSyn pathology in brains of M83$^{+/+}$ mice.

S4. Heatmaps depicting the relative expression of select transcripts in PD-patient derived NCBI GEO microarray datasets. (A-B) Heatmaps depicting the expression of select transcripts across PD microarray datasets (with Log2FC and p-value), which were significantly upregulated (in A) or downregulated (in B) in the ST dataset from the animal model. The microarray datasets and brain regions examined include: 1) GSE7621 (*substantia nigra-SN*; Controls, n=9; PD, n=16), 2) GSE43490 (*SN, dorsal motor nucleus of vagus- dmX and locus coeruleus-LC*; Controls, n=5-7; PD, n=8), 3) GSE20146 (*globus pallidus, interna-GPi*; Controls, n=10; PD, n=10) and 4) GSE26927 (*SN*; Controls, n=7; PD, n=12). Also see Table S6.

S5. ST maps and IF detection of ROCK2 in brains of M83$^{+/+}$ mice. (A) Violin plot and representative spatial maps depicting the relative abundance of *Rock2* in brains of M83$^{+/+}$ mice. **(B)** Representative (20X) images showing ROCK2 IF in the gigantocellular nuclei (GRN, in pons), periaqueductal grey (PAG, in midbrain), deep cerebellar nuclei (DCN, in cerebellum) and mediodorsal nuclei (MD, in thalamus). The inset show 63X magnified views from the regions. Scale bar= 50 µm. **(C)** Bar graphs depicting quantification of ROCK2 IF in GRN, PAG, DCN and DM of the experimental cohorts, as indicated. Error bars depict Mean IF intensity ± SD as % of

cells in total area (PBS, n=2; DPI-45, n=3 and DPI-75, n=3; See Methods). Statistics in S5C: Kruskal-Wallis ANOVA (p-values on graphs), Dunn's multiple comparisons (non-significant). **S6. Case by case plots for p-aSyn (S129) and CREBBP/CBP IHC in post-mortem human brain sections. (A-B)** Bar graphs depicting quantification of p-aSyn, S129 and CREBBP/CBP IHC in the SN*pc* (in A) and PAG (in B) of 6 controls and 10 PD cases examined in the study. Graphs display Mean ± SEM of Lewy bodies per mm^2 and Mean % ± SEM of CREBBP/CBP immunopositive cells in the region (n=6 20X views/region/case or control). Notice the relatively higher detection of CREBBP in PD cases with diffuse LB + dementia (Table S1). Also see Fig. 6A-D.

Figure S1

A)

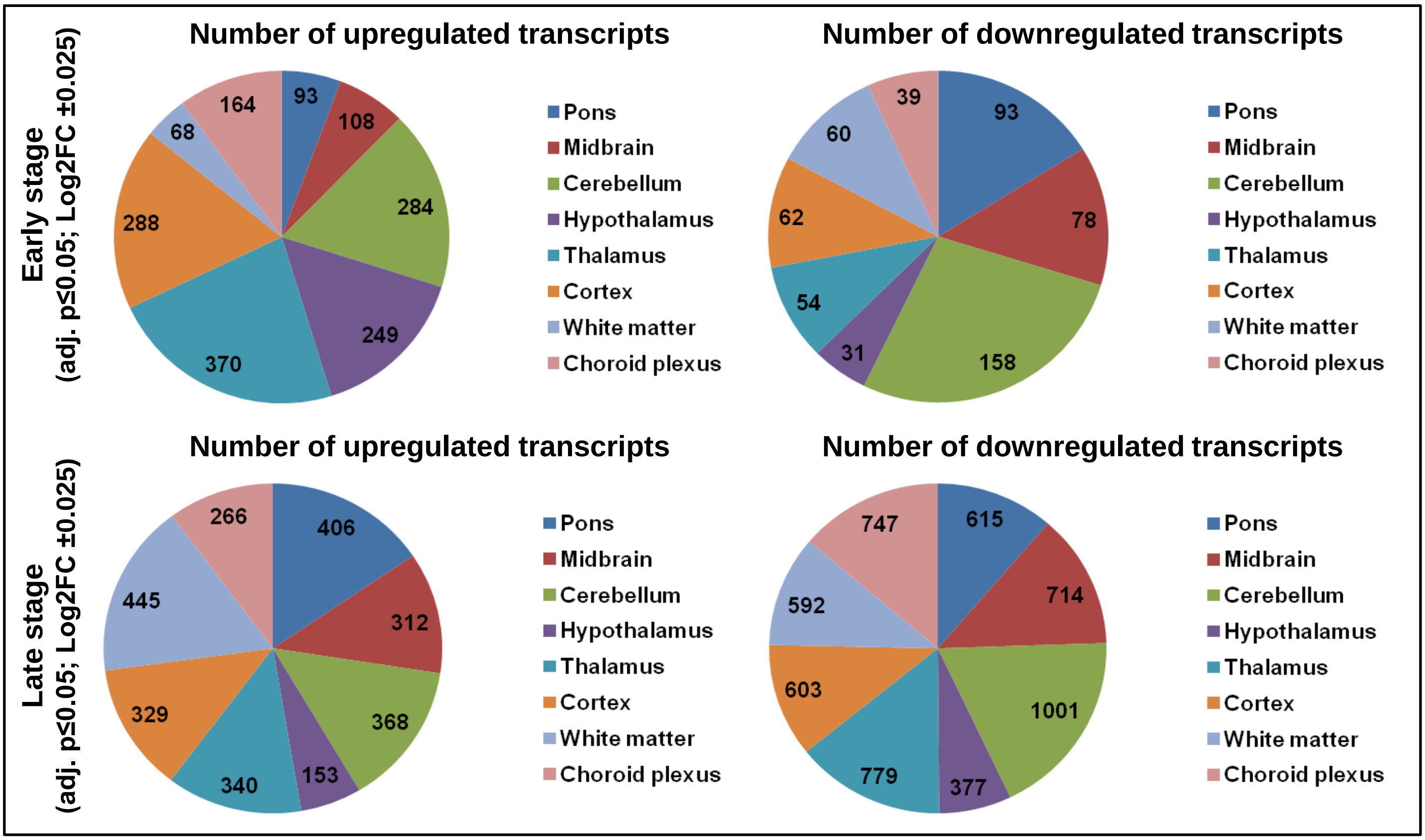
Number of upregulated transcripts
Number of downregulated transcripts
Early stage
(adj. p≤0.05; Log2FC ±0.025)
93
108
284
249
370
288
68
164
93
78
158
31
54
62
60
39
Pons
Midbrain
Cerebellum
Hypothalamus
Thalamus
Cortex
White matter
Choroid plexus
Late stage
(adj. p≤0.05; Log2FC ±0.025)
406
312
368
153
340
329
445
266
615
714
1001
377
779
603
592
747

B)

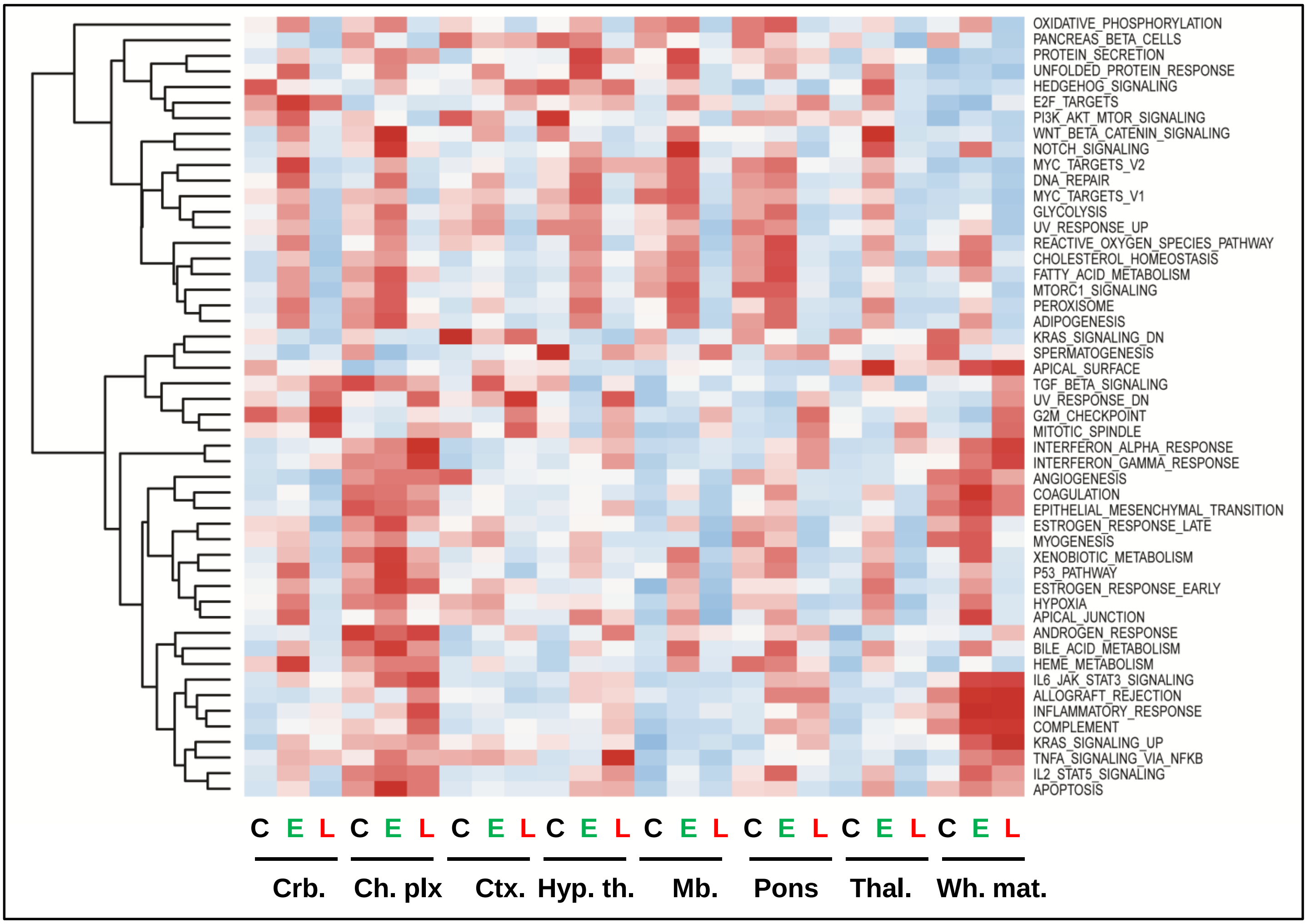
OXIDATIVE_PHOSPHORYLATION
PANCREAS_BETA_CELLS
PROTEIN_SECRETION
UNFOLDED_PROTEIN_RESPONSE
HEDGEHOG_SIGNALING
E2F_TARGETS
PI3K_AKT_MTOR_SIGNALING
WNT_BETA_CATENIN_SIGNALING
NOTCH_SIGNALING
MYC_TARGETS_V2
DNA_REPAIR
MYC_TARGETS_V1
GLYCOLYSIS
UV_RESPONSE_UP
REACTIVE_OXYGEN_SPECIES_PATHWAY
CHOLESTEROL_HOMEOSTASIS
FATTY_ACID_METABOLISM
MTORC1_SIGNALING
PEROXISOME
ADIPOGENESIS
KRAS_SIGNALING_DN
SPERMATOGENESIS
APICAL_SURFACE
TGF_BETA_SIGNALING
UV_RESPONSE_DN
G2M_CHECKPOINT
MITOTIC_SPINDLE
INTERFERON_ALPHA_RESPONSE
INTERFERON_GAMMA_RESPONSE
ANGIOGENESIS
COAGULATION
EPITHELIAL_MESENCHYMAL_TRANSITION
ESTROGEN_RESPONSE_LATE
MYOGENESIS
XENOBIOTIC_METABOLISM
P53_PATHWAY
ESTROGEN_RESPONSE_EARLY
HYPOXIA
APICAL_JUNCTION
ANDROGEN_RESPONSE
BILE_ACID_METABOLISM
HEME_METABOLISM
IL6_JAK_STAT3_SIGNALING
ALLOGRAFT_REJECTION
INFLAMMATORY_RESPONSE
COMPLEMENT
KRAS_SIGNALING_UP
TNFA_SIGNALING_VIA_NFKB
IL2_STAT5_SIGNALING
APOPTOSIS
C E L C E L C E L C E L C E L C E L C E L C E L
Crb.
Ch. plx
Ctx.
Hyp. th.
Mb.
Pons
Thal.
Wh. mat.

Figure S2

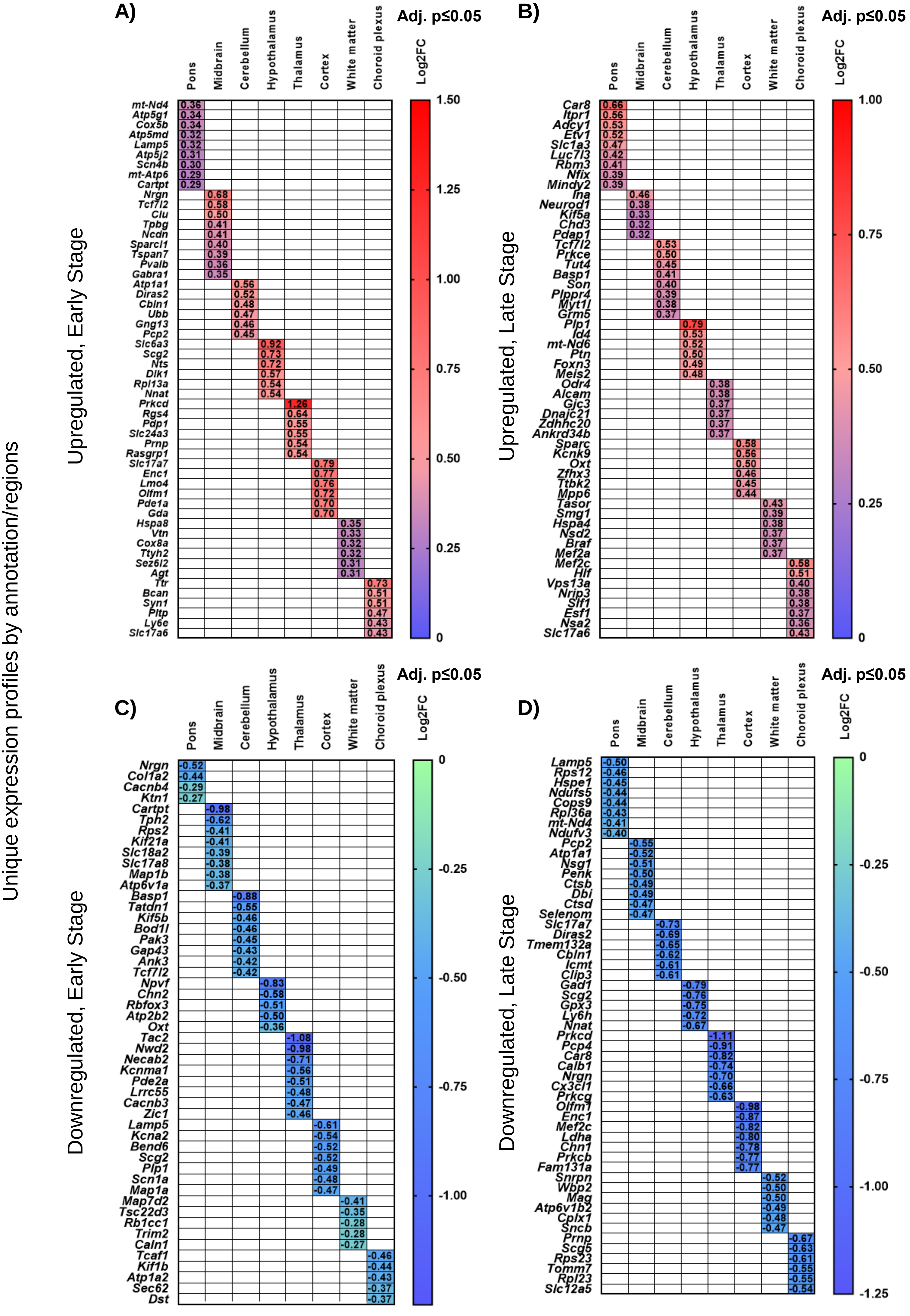
Unique expression profiles by annotation/regions
A)
Upregulated, Early Stage
Pons
Midbrain
Cerebellum
Hypothalamus
Thalamus
Cortex
White matter
Choroid plexus
Adj. p≤0.05
Log2FC
1.50
1.25
1.00
0.75
0.50
0.25
0
mt-Nd4 0.36
Atp5g1 0.34
Cox5b 0.34
Atp5md 0.32
Lamp5 0.32
Atp5j2 0.31
Scn4b 0.30
mt-Atp6 0.29
Cartpt 0.29
Nrgn 0.68
Tcf7l2 0.58
Clu 0.50
Tpbg 0.41
Ncdn 0.41
Sparcl1 0.40
Tspan7 0.39
Pvalb 0.36
Gabra1 0.35
Atp1a1 0.56
Diras2 0.52
Cbln1 0.48
Ubb 0.47
Gng13 0.46
Pcp2 0.45
Slc6a3 0.92
Scg2 0.73
Nts 0.72
Dlk1 0.57
Rpl13a 0.54
Nnat 0.54
Prkcd 1.26
Rgs4 0.64
Pdp1 0.55
Slc24a3 0.55
Prnp 0.54
Rasgrp1 0.54
Slc17a7 0.79
Enc1 0.77
Lmo4 0.76
Olfm1 0.72
Pde1a 0.70
Gda 0.70
Hspa8 0.35
Vtn 0.33
Cox8a 0.32
Ttyh2 0.32
Sez6l2 0.31
Agt 0.31
Ttr 0.73
Bcan 0.51
Syn1 0.51
Pltp 0.47
Ly6e 0.43
Slc17a6 0.43
B)
Upregulated, Late Stage
Pons
Midbrain
Cerebellum
Hypothalamus
Thalamus
Cortex
White matter
Choroid plexus
Adj. p≤0.05
Log2FC
1.00
0.75
0.50
0.25
0
Car8 0.66
Itpr1 0.56
Adcy1 0.53
Etv1 0.52
Slc1a3 0.47
Luc7l3 0.42
Rbm3 0.41
Nfix 0.39
Mindy2 0.39
Ina 0.46
Neurod1 0.38
Kif5a 0.33
Chd3 0.32
Pdap1 0.32
Tcf7l2 0.53
Prkce 0.50
Tut4 0.45
Basp1 0.41
Son 0.40
Plppr4 0.39
Myt1l 0.38
Grm5 0.37
Plp1 0.79
Id4 0.53
mt-Nd6 0.52
Ptn 0.50
Foxn3 0.49
Meis2 0.48
Odr4 0.38
Alcam 0.38
Gjc3 0.37
Dnajc21 0.37
Zdhhc20 0.37
Ankrd34b 0.37
Sparc 0.58
Kcnk9 0.56
Oxt 0.50
Zfhx3 0.46
Ttbk2 0.45
Mpp6 0.44
Tasor 0.43
Smg1 0.39
Hspa4 0.38
Nsd2 0.37
Braf 0.37
Mef2a 0.37
Mef2c 0.58
Hlf 0.51
Vps13a 0.40
Nrip3 0.38
Slf1 0.38
Esf1 0.37
Nsa2 0.36
Slc17a6 0.43
C)
Downregulated, Early Stage
Pons
Midbrain
Cerebellum
Hypothalamus
Thalamus
Cortex
White matter
Choroid plexus
Adj. p≤0.05
Log2FC
0
-0.25
-0.50
-0.75
-1.00
Nrgn -0.52
Col1a2 -0.44
Cacnb4 -0.29
Ktn1 -0.27
Cartpt -0.98
Tph2 -0.62
Rps2 -0.41
Kif21a -0.41
Slc18a2 -0.39
Slc17a8 -0.38
Map1b -0.38
Atp6v1a -0.37
Basp1 -0.88
Tatdn1 -0.55
Kif5b -0.46
Bod1l -0.46
Pak3 -0.45
Gap43 -0.43
Ank3 -0.42
Tcf7l2 -0.42
Npvf -0.83
Chn2 -0.58
Rbfox3 -0.51
Atp2b2 -0.50
Oxt -0.36
Tac2 -1.08
Nwd2 -0.98
Necab2 -0.71
Kcnma1 -0.56
Pde2a -0.51
Lrrc55 -0.48
Cacnb3 -0.47
Zic1 -0.46
Lamp5 -0.61
Kcna2 -0.54
Bend6 -0.52
Scg2 -0.52
Plp1 -0.49
Scn1a -0.48
Map1a -0.47
Map7d2 -0.41
Tsc22d3 -0.35
Rb1cc1 -0.28
Trim2 -0.28
Caln1 -0.27
Tcaf1 -0.46
Kif1b -0.44
Atp1a2 -0.43
Sec62 -0.37
Dst -0.37
D)
Downregulated, Late Stage
Pons
Midbrain
Cerebellum
Hypothalamus
Thalamus
Cortex
White matter
Choroid plexus
Adj. p≤0.05
Log2FC
0
-0.25
-0.50
-0.75
-1.00
-1.25
Lamp5 -0.50
Rps12 -0.46
Hspe1 -0.45
Ndufs5 -0.44
Cops9 -0.44
Rpl36a -0.43
mt-Nd4 -0.41
Ndufv3 -0.40
Pcp2 -0.55
Atp1a1 -0.52
Nsg1 -0.51
Penk -0.50
Ctsb -0.49
Dbi -0.49
Ctsd -0.47
Selenom -0.47
Slc17a7 -0.73
Diras2 -0.69
Tmem132a -0.65
Cbln1 -0.62
Icmt -0.61
Clip3 -0.61
Gad1 -0.79
Scg2 -0.76
Gpx3 -0.75
Ly6h -0.72
Nnat -0.67
Prkcd -1.11
Pcp4 -0.91
Car8 -0.82
Calb1 -0.74
Nrgn -0.70
Cx3cl1 -0.66
Prkcg -0.63
Olfm1 -0.98
Enc1 -0.87
Mef2c -0.82
Ldha -0.80
Chn1 -0.78
Prkcb -0.77
Fam131a -0.77
Snrpn -0.52
Wbp2 -0.50
Mag -0.50
Atp6v1b2 -0.49
Cplx1 -0.48
Sncb -0.47
Prnp -0.67
Scg5 -0.63
Rps23 -0.61
Tomm7 -0.55
Rpl23 -0.55
Slc12a5 -0.54

Figure S3

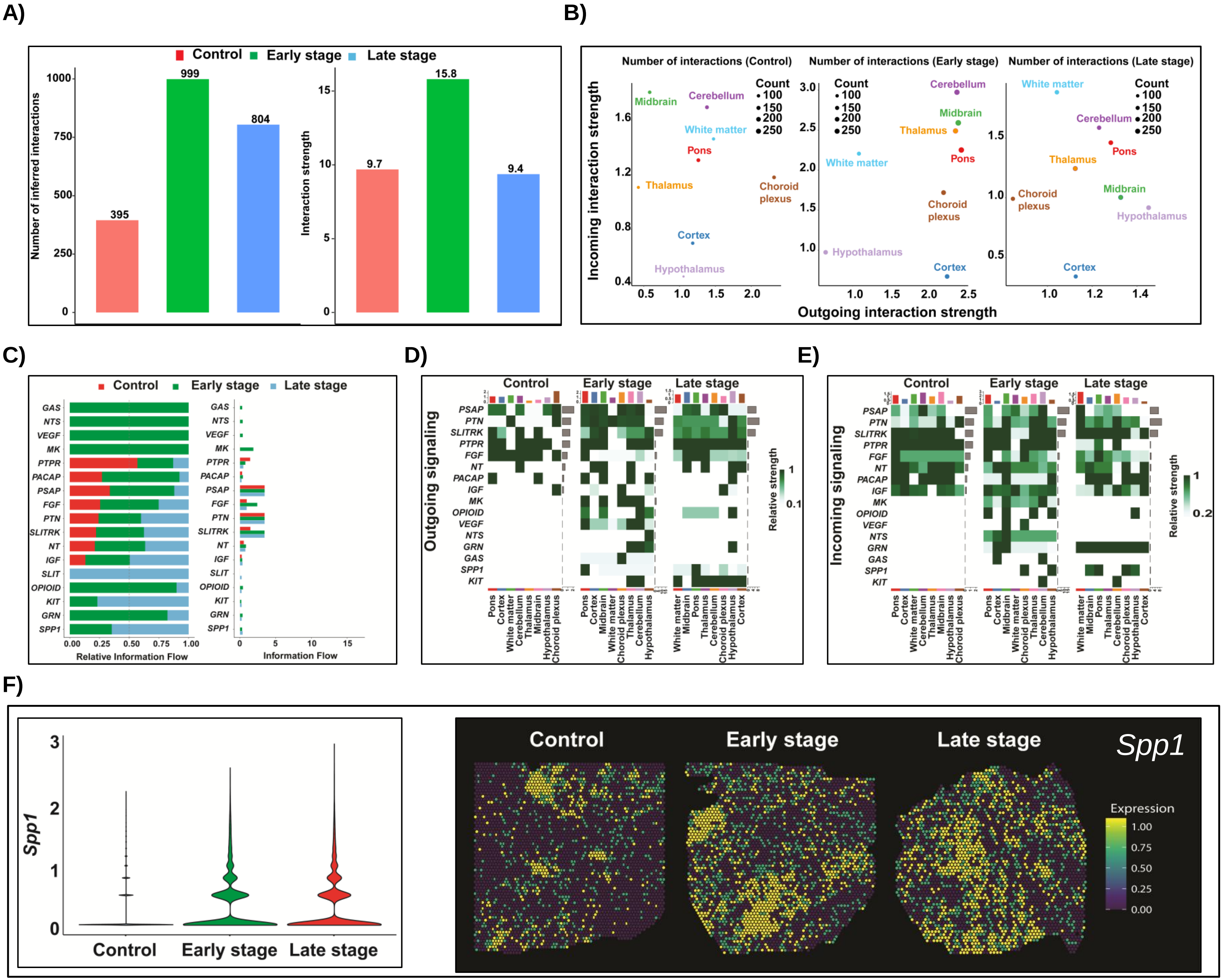

A)
Control
Early stage
Late stage
Number of inferred interactions
395
999
804
Interaction strength
9.7
15.8
9.4
B)
Number of interactions (Control)
Number of interactions (Early stage)
Number of interactions (Late stage)
Incoming interaction strength
Outgoing interaction strength
Count
100
150
200
250
Midbrain
Cerebellum
White matter
Pons
Thalamus
Choroid plexus
Cortex
Hypothalamus
C)
Relative Information Flow
Information Flow
GAS
NTS
VEGF
MK
PTPR
PACAP
PSAP
FGF
PTN
SLITRK
NT
IGF
SLIT
OPIOID
KIT
GRN
SPP1
D)
Outgoing signaling
Relative strength
E)
Incoming signaling
F)
Spp1
Expression

Figure S4

A) B)

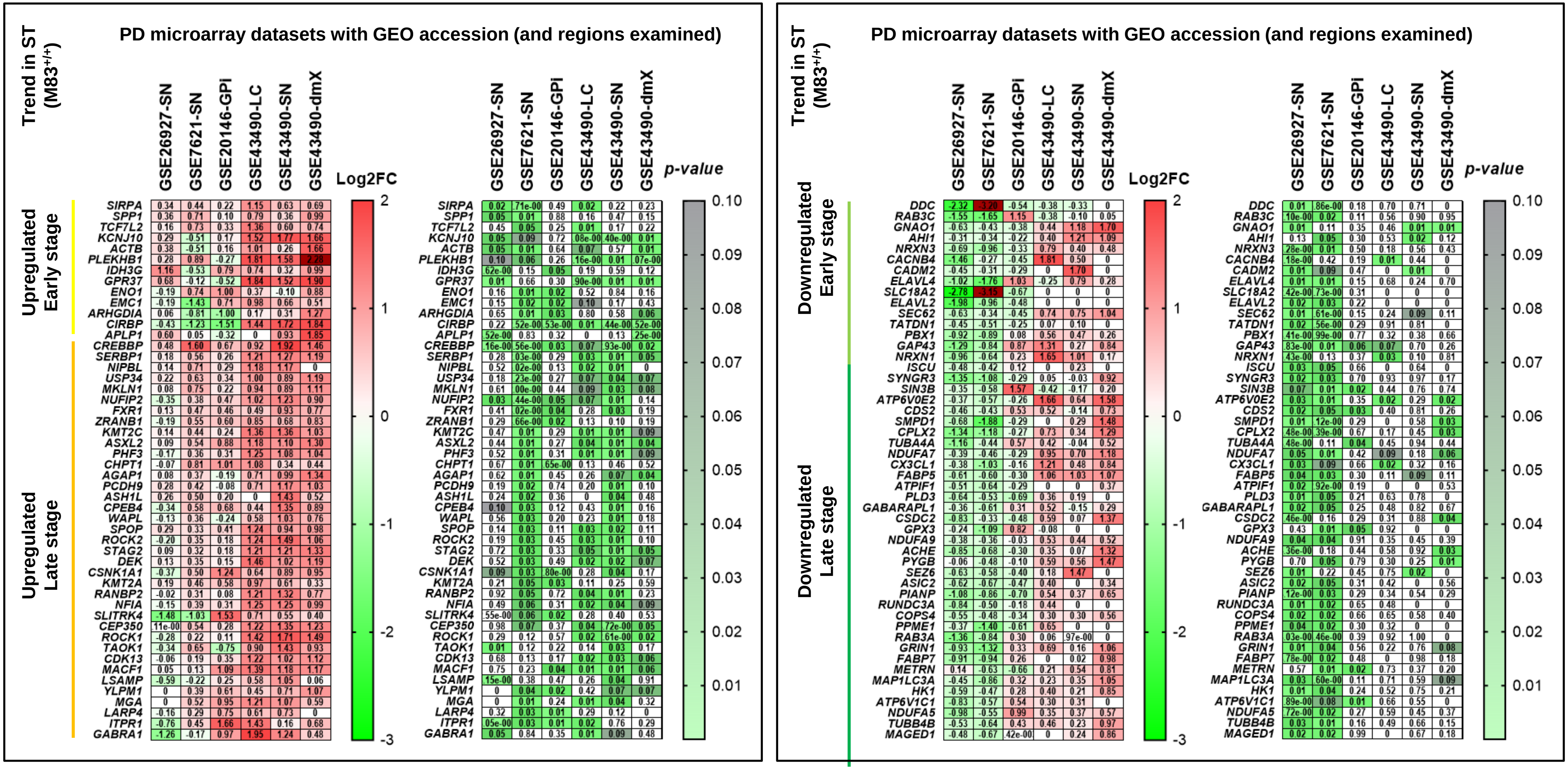

PD microarray datasets with GEO accession (and regions examined)
Trend in ST (M83+/+)
GSE26927-SN
GSE7621-SN
GSE20146-GPi
GSE43490-LC
GSE43490-SN
GSE43490-dmX
Log2FC
p-value
Upregulated Early stage
Upregulated Late stage
SIRPA SPP1 TCF7L2 KCNJ10 ACTB PLEKHB1 IDH3G GPR37 ENO1 EMC1 ARHGDIA CIRBP APLP1 CREBBP SERBP1 NIPBL USP34 MKLN1 NUFIP2 FXR1 ZRANB1 KMT2C ASXL2 PHF3 CHPT1 AGAP1 PCDH9 ASH1L CPEB4 WAPL SPOP ROCK2 STAG2 DEK CSNK1A1 KMT2A RANBP2 NFIA SLITRK4 CEP350 ROCK1 TAOK1 CDK13 MACF1 LSAMP YLPM1 MGA LARP4 ITPR1 GABRA1
Downregulated Early stage
Downregulated Late stage
DDC RAB3C GNAO1 AHI1 NRXN3 CACNB4 CADM2 ELAVL4 SLC18A2 ELAVL2 SEC62 TATDN1 PBX1 GAP43 NRXN1 ISCU SYNGR3 SIN3B ATP6V0E2 CDS2 SMPD1 CPLX2 TUBA4A NDUFA7 CX3CL1 FABP5 ATPIF1 PLD3 GABARAPL1 CSDC2 GPX3 NDUFA9 ACHE PYGB SEZ6 ASIC2 PIANP RUNDC3A COPS4 PPME1 RAB3A GRIN1 FABP7 METRN MAP1LC3A HK1 ATP6V1C1 NDUFA5 TUBB4B MAGED1

Figure S5

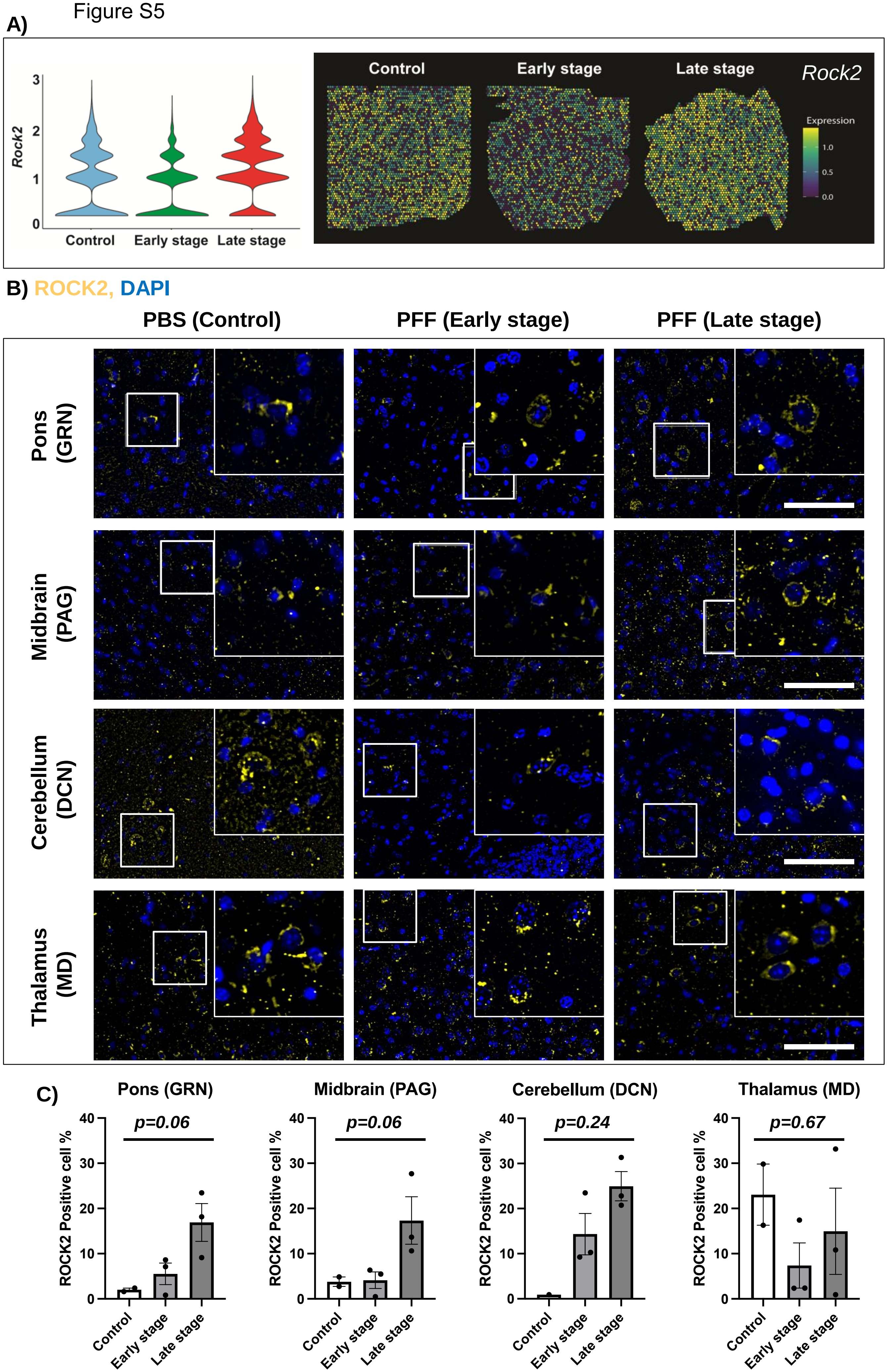
A)
Rock2
3
2
1
0
Control
Early stage
Late stage
Control
Early stage
Late stage
Rock2
Expression
1.0
0.5
0.0
B) ROCK2, DAPI
PBS (Control)
PFF (Early stage)
PFF (Late stage)
Pons (GRN)
Midbrain (PAG)
Cerebellum (DCN)
Thalamus (MD)
C)
Pons (GRN)
Midbrain (PAG)
Cerebellum (DCN)
Thalamus (MD)
ROCK2 Positive cell %
40
30
20
10
0
p=0.06
p=0.06
p=0.24
p=0.67
Control
Early stage
Late stage

Figure S6

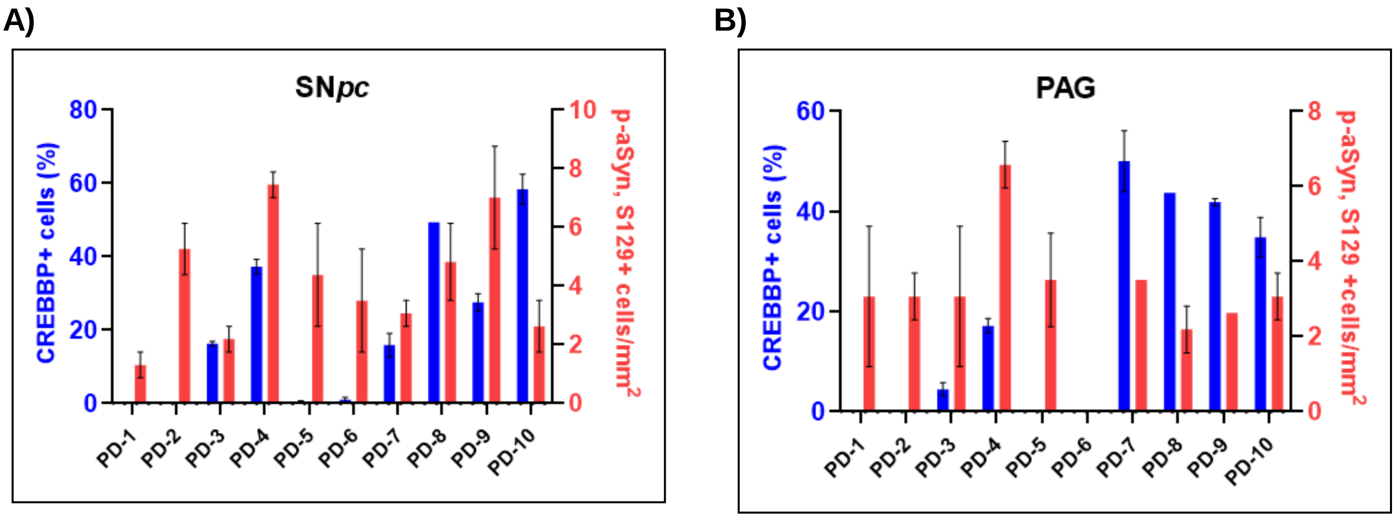
A)
SNpc
CREBBP+ cells (%)
p-aSyn, S129+ cells/mm²
0
20
40
60
80
0
2
4
6
8
10
PD-1
PD-2
PD-3
PD-4
PD-5
PD-6
PD-7
PD-8
PD-9
PD-10
B)
PAG
CREBBP+ cells (%)
p-aSyn, S129 +cells/mm²
0
20
40
60
0
2
4
6
8
PD-1
PD-2
PD-3
PD-4
PD-5
PD-6
PD-7
PD-8
PD-9
PD-10

Data S1. User guide to the ST datasets

Introduction to the Synucleinopathy M83 model Spatial Database

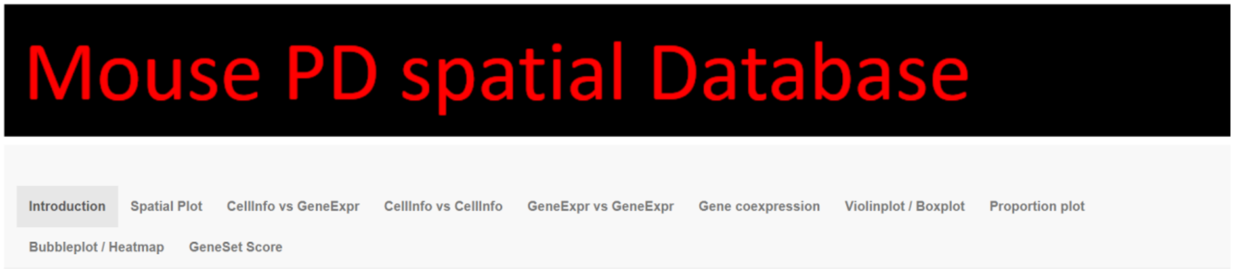

The Mouse PD spatial database is designed to help researchers and clinicians explore gene expression profiles in the context of Parkinson’s disease mouse models. Here’s a practical guide on how to navigate and utilize the database effectively:

Accessing the Database

https://dreamapp.biomed.au.dk/PD_spatial_mouse_DB/

Navigating the Interface: Use the tabs at the top of the page to switch between different sections of the database: Introduction, Spatial Plot, CellInfo vs GeneExpr, CellInfo vs CellInfo, GeneExpr vs GeneExpr, Gene coexpression, Violinplot/ Boxplot, Proportion plot, Bubbleplot/ Heatmap, GeneSet Score.

Data Visualization:

1. Spatial Plots
2. UMAPs
3. Violin and Box plots
4. Cell Proportions
5. Heatmaps and Bubble plots
6. Gene set scores (HALLMARK/KEGG)

1. How to Use the Spatial Plot Tab

The Spatial Plot tab in the Mouse Parkinson's Disease (PD) spatial database allows users to explore the genes that exhibit significant changes in expression levels across different brain regions and different time points (early PD or late PD vs. sham conditions). Here's a detailed guide on how to use this feature:

1.1. Accessing Spatial gene expression

1.1.1. Select the Spatial Plot Tab: Click on the "Spatial Plot" tab on the top navigation bar to open the differentially expressed genes analysis interface.

1.2 Searching for a Specific Gene

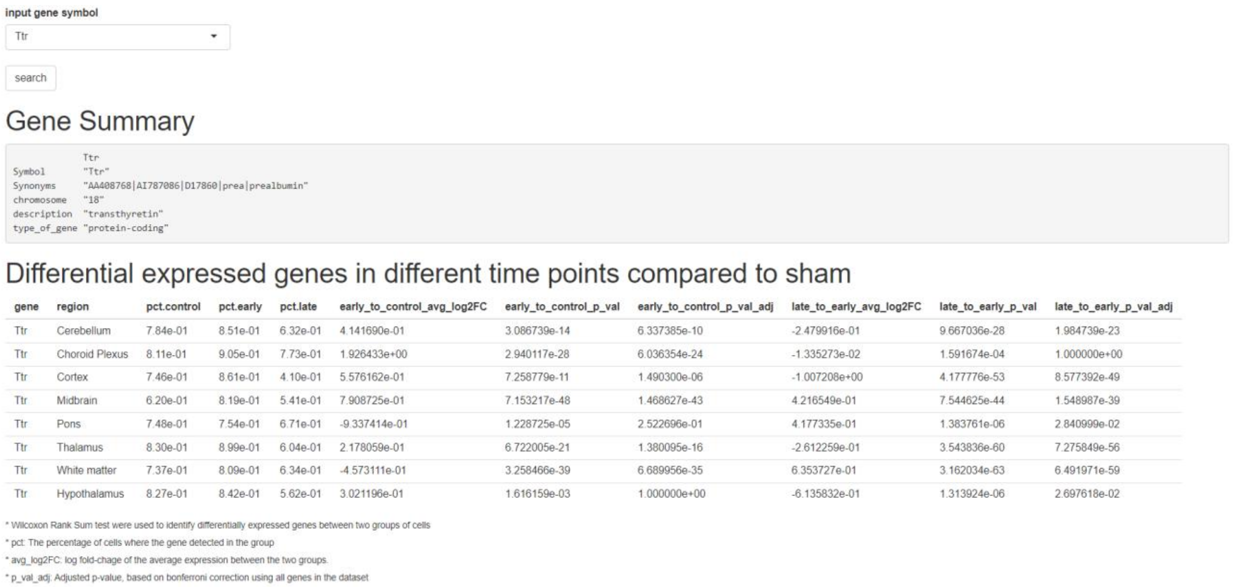
input gene symbol
Ttr
search

Gene Summary

```
             Ttr
Symbol       "Ttr"
Synonyms     "AA408768|AI787086|D17860|prea|prealbumin"
chromosome   "18"
description  "transthyretin"
type_of_gene "protein-coding"
```

Differential expressed genes in different time points compared to sham

gene	region	pct.control	pct.early	pct.late	early_to_control_avg_log2FC	early_to_control_p_val	early_to_control_p_val_adj	late_to_early_avg_log2FC	late_to_early_p_val	late_to_early_p_val_adj
Ttr	Cerebellum	7.84e-01	8.51e-01	6.32e-01	4.141690e-01	3.086739e-14	6.337385e-10	-2.479916e-01	9.667036e-28	1.984739e-23
Ttr	Choroid Plexus	8.11e-01	9.05e-01	7.73e-01	1.926433e+00	2.940117e-28	6.036354e-24	-1.335273e-02	1.591674e-04	1.000000e+00
Ttr	Cortex	7.46e-01	8.61e-01	4.10e-01	5.576162e-01	7.258779e-11	1.490300e-06	-1.007208e+00	4.177776e-53	8.577392e-49
Ttr	Midbrain	6.20e-01	8.19e-01	5.41e-01	7.908725e-01	7.153217e-48	1.468627e-43	4.216549e-01	7.544625e-44	1.548987e-39
Ttr	Pons	7.48e-01	7.54e-01	6.71e-01	-9.337414e-01	1.228725e-05	2.522696e-01	4.177335e-01	1.383761e-06	2.840999e-02
Ttr	Thalamus	8.30e-01	8.99e-01	6.04e-01	2.178059e-01	6.722005e-21	1.380095e-16	-2.612259e-01	3.543836e-60	7.275849e-56
Ttr	White matter	7.37e-01	8.09e-01	6.34e-01	-4.573111e-01	3.258466e-39	6.689956e-35	6.353727e-01	3.162034e-63	6.491971e-59
Ttr	Hypothalamus	8.27e-01	8.42e-01	5.62e-01	3.021196e-01	1.616159e-03	1.000000e+00	-6.135832e-01	1.313924e-06	2.697618e-02

* Wilcoxon Rank Sum test were used to identify differentially expressed genes between two groups of cells
* pct: The percentage of cells where the gene detected in the group
* avg_log2FC: log fold-chage of the average expression between the two groups.
* p_val_adj: Adjusted p-value, based on bonferroni correction using all genes in the dataset

1.2.1. Input Gene Symbol:

- Type the gene symbol of interest in the input box (e.g., "Ttr").
- Click the "search" button to retrieve the gene expression data.

1.2.2. Gene Summary:

- After searching, a summary of the gene will be displayed, including its symbol, synonyms, chromosome location, description, and type

1.2.3. View Results:

- The results will be displayed in a table, showing various columns such as:
  - **Gene**: The selected gene, in this example the transthyretin (Ttr) gene
  - **Region**: The specific brain region selected.
  - **Pct.control**: The percentage of cells where the gene was detected in the control group
  - **Pct.early**: The percentage of cells expressing the gene in the early PD group
  - **Pct.late**: The percentage of cells expressing the gene in the late PD group

- **avg_log2FC**: The average log2 fold change in expression in the different conditions (early_to_control and late_to_early).
- **P_val**: The p-value indicating the statistical significance of the expression change.
- **P_val_adj**: The adjusted p-value for multiple comparisons.

1.3. Visualizing Spatial Gene Expression

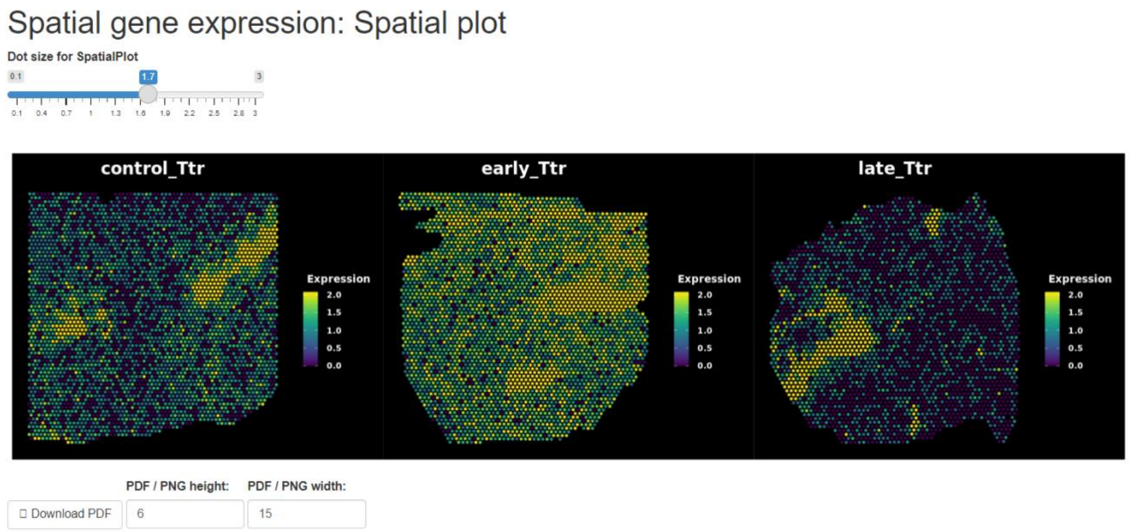

1.3.1. Spatial gene expression: Spatial Plot:

- This section provides a visual representation of the gene expression in spatial context. You can see the selected gene expression in different time points and different brain regions (vs. sham), together with their corresponding spatial plots
- Adjust the dot size using the slider to change the visualization resolution.
- Download the spatial plot as a PDF or PNG file by setting the desired dimensions and clicking the download buttons.

2. Analyzing Gene Expression on Reduced Dimensions

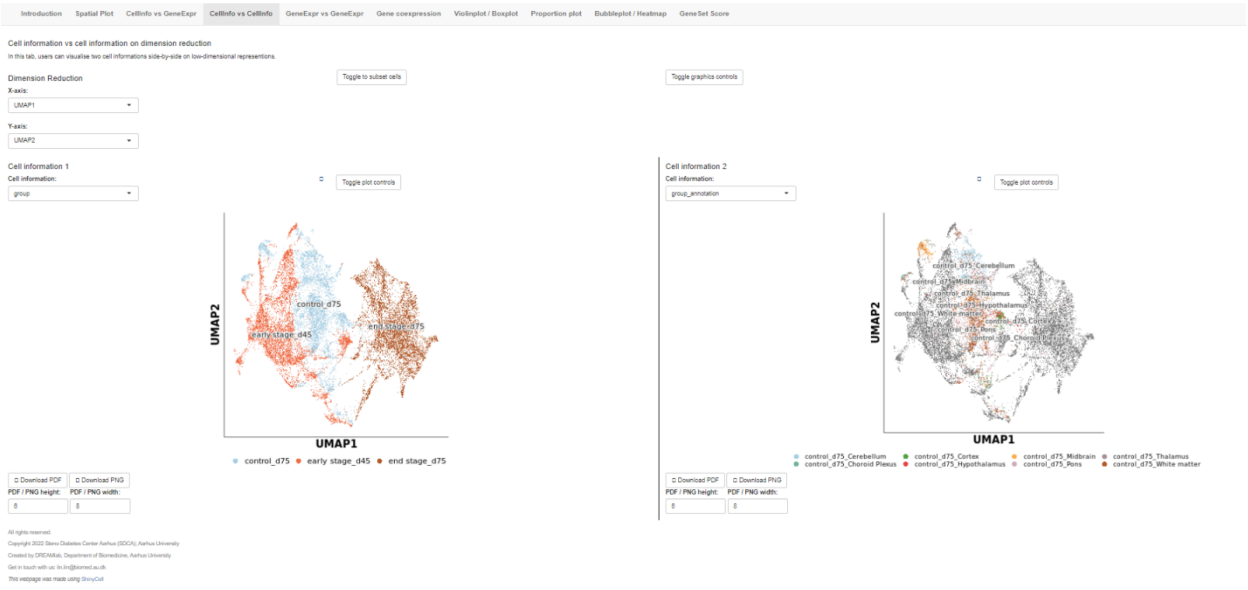

2.1. Dimension Reduction Plot – CellInfo vs GeneExpr

- Use this section to visualize gene expression on reduced dimensions (e.g., UMAP);
- Select the axes for dimension reduction (e.g., UMAP1 and UMAP2);
- Choose the primary cell information you are interested;
- Click on “Toggle to subset cells” to toggle between different spot information and gene expression views. Here you can select which cells to show by selecting “Cell information to subset”;
- On the right panel select the gene you would like to visualize on the UMAP plot;
- Download the plots as PDF or PNG files.

Data S1. User guide to the ST datasets

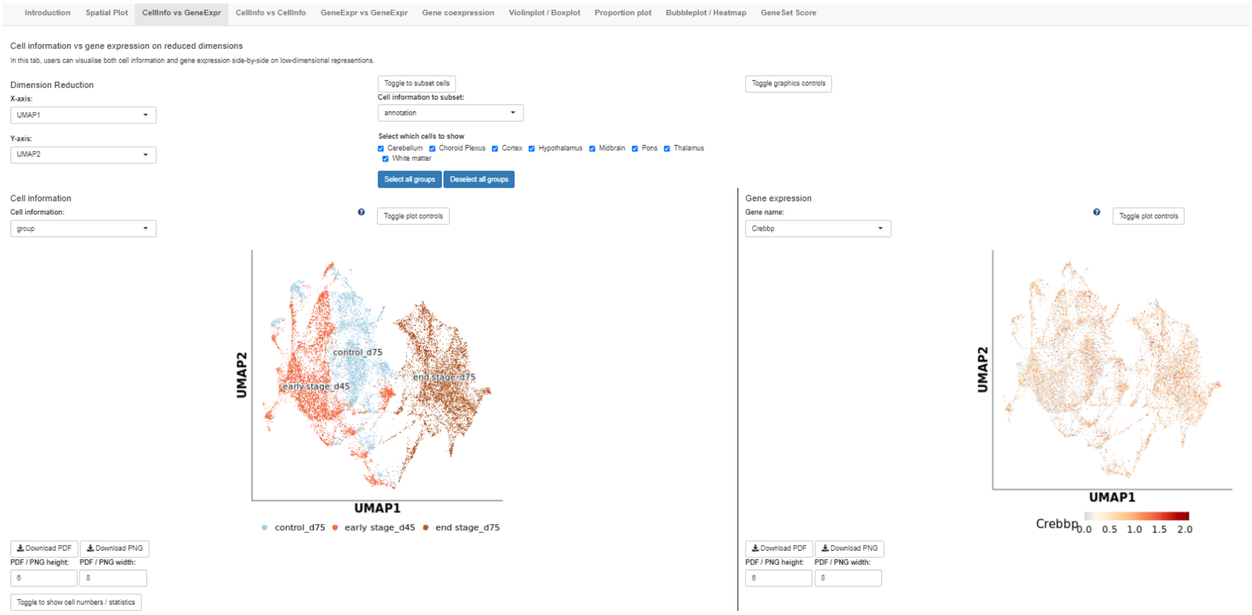

2.2. Dimension Reduction Plot –CellInfo vs CellInfo

- Similarly to above, use this section to visualize cell information on reduced dimensions (e.g., UMAP);
- Select the axes for dimension reduction (e.g., UMAP1 and UMAP2);
- Toggle between different spot information and cell information views;
- Download the plots as PDF or PNG files.

2.3. Co-expression of Two Genes

2.3.1. Dimension Reduction Plot –GeneExpr vs GeneExpr:

- Use this section to visualize gene co-expression on reduced dimensions (e.g., UMAP);
- Select the axes for dimension reduction (e.g., UMAP1 and UMAP2);
- Toggle between different spot information and gene expression views;
- Download the plots as PDF or PNG files.

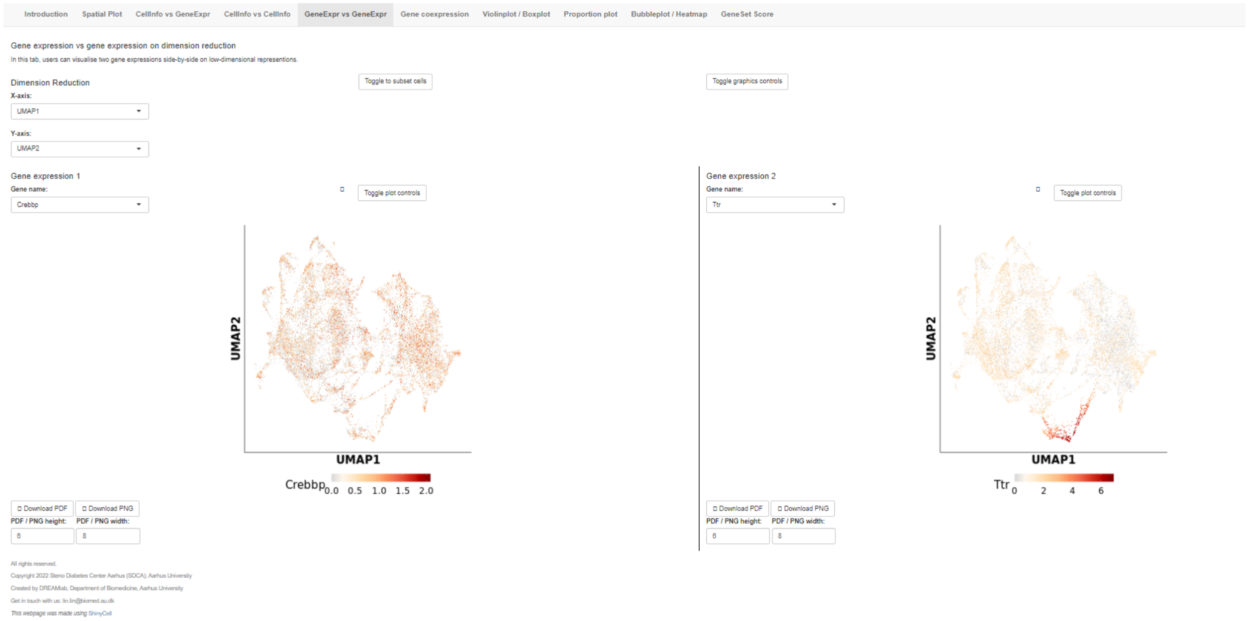

2.3.2. Co-expression Plot:

- This section allows you to analyze the co-expression of two genes on reduced dimensions;
- Select the genes to be analyzed (e.g., "Crebbp" and "Ttr");
- The plot shows the spatial distribution of cells expressing both genes;
- Download the co-expression plot as a PDF or PNG file.

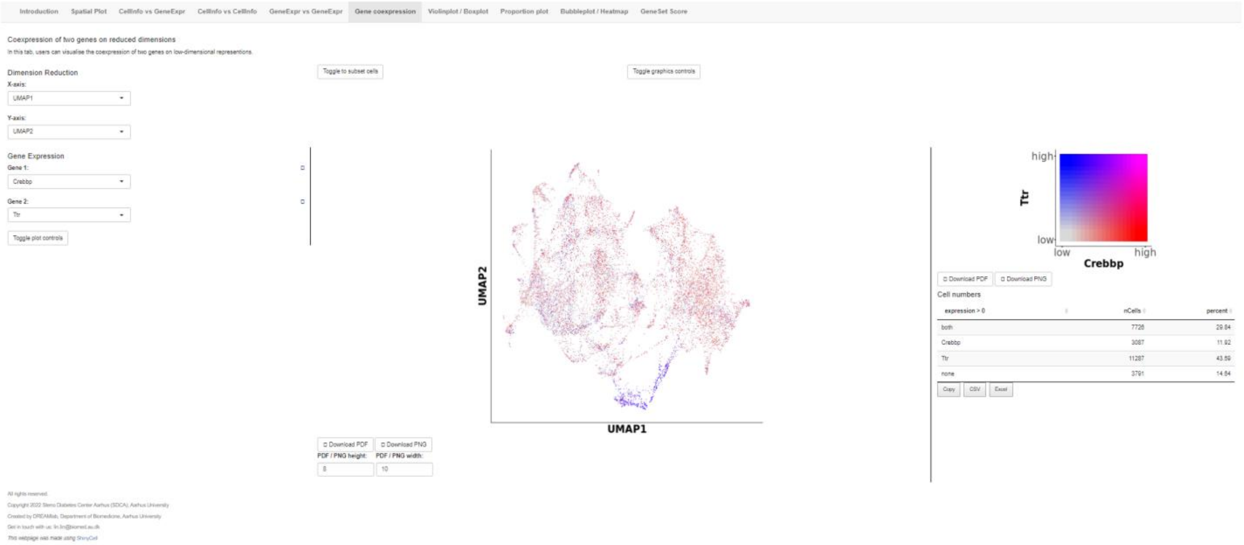

3. Visualizing Gene Expression with Violin and Box Plots

<u>3.1. Violin/Box Plot</u>:

- This section provides a visual representation of gene expression distribution using violin or box plots;
- Select the X-axis and Y-axis parameters, and choose the plot type (violin or boxplot);
- Select “Toggle to subset cells” to choose the cell information to subset; Choose between group or annotation to select specific brain regions
- Toggle between showing data points and adjusting graphics controls;
- Download the plots as PDF or PNG files.

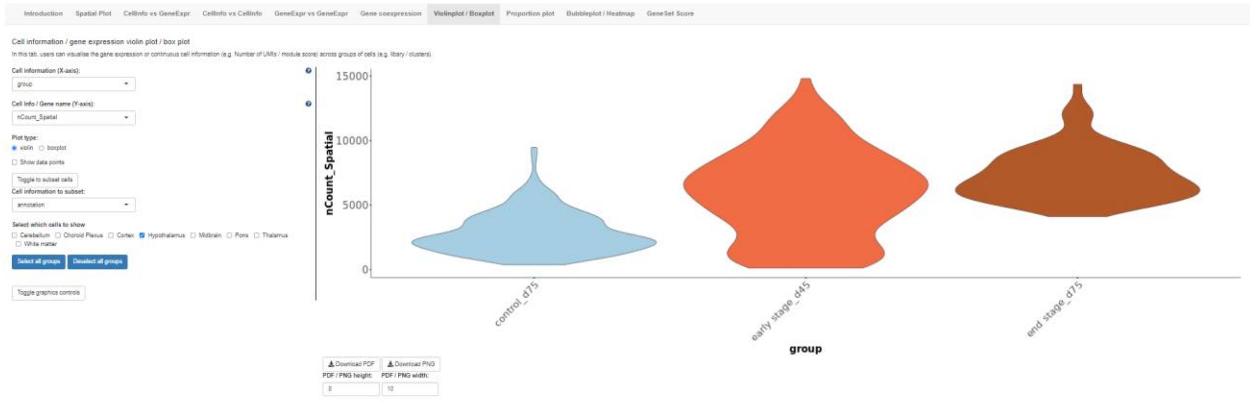

4. Generating cell proportion and cell number plots

<u>4.1. Cell proportion (%)</u>:

- This section allows you to visualize the cell proportion (%) in each disease stage and brain region;
- Select the X-axis and Y-axis parameters, and choose the plot value (proportion or cell number);
- Select “Toggle to subset cells” to choose the cell information to subset; Choose between group or annotation to select specific brain regions;
- Toggle graphic controls to select plot size and font size for better visualization;
- Download the plots as PDF or PNG files.

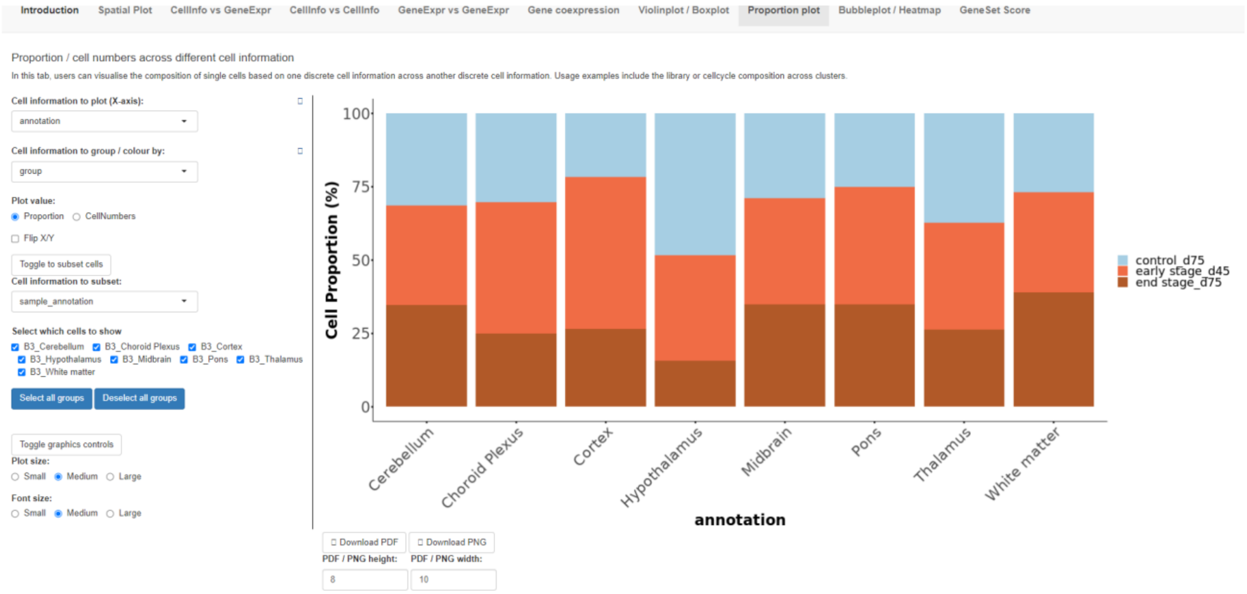

5. Creating Gene Expression Bubbleplots/Heatmaps

<u>5.1. Bubbleplot/Heatmap</u>:

- This section allows you to visualize gene expression patterns of multiple genes.
- Enter a list of gene names and select the grouping parameter (e.g., group).
- Choose the plot type (bubbleplot or heatmap).
- Cluster rows (genes) and columns (samples) for better visualization.
- Download the plots as PDF or PNG files.

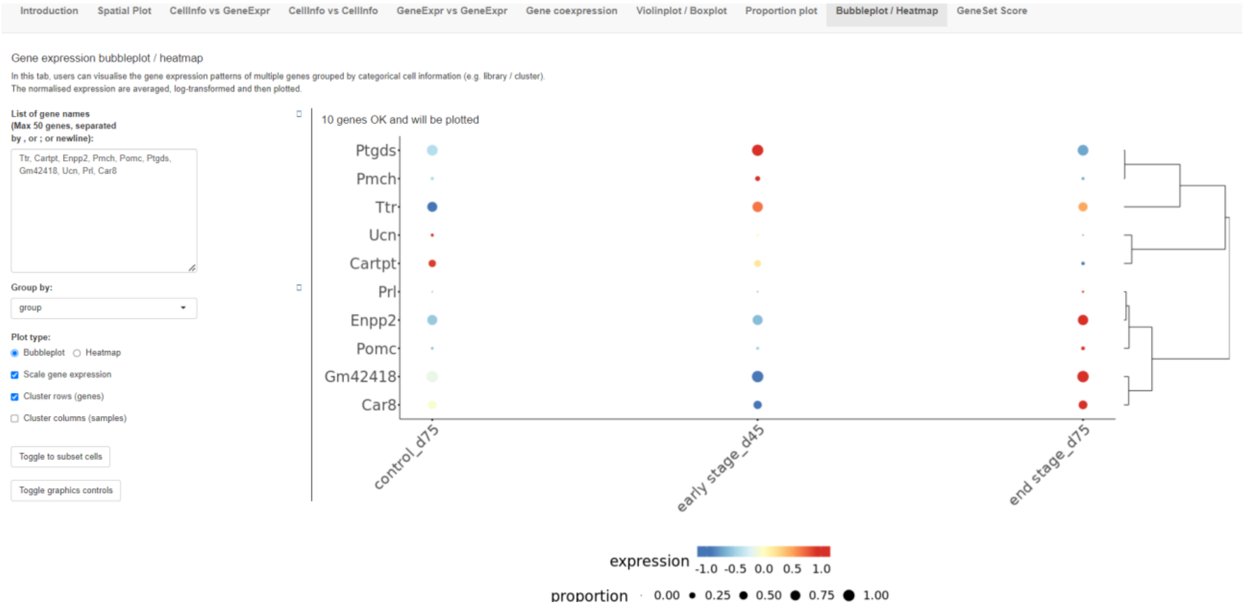

6. How to Use GeneSet Score Tab

The GeneSet Score tab in the Mouse PD spatial database allows users to visualize gene set scores across different brain regions and conditions. This feature helps in understanding the collective behavior of groups of genes involved in specific biological processes or pathways. Here’s a detailed guide on how to use this feature effectively:

6.1. Accessing GeneSet Scores

Click on the "GeneSet Score" tab on the top navigation bar to open the gene set score analysis interface.

6.2. Visualizing GeneSet Scores

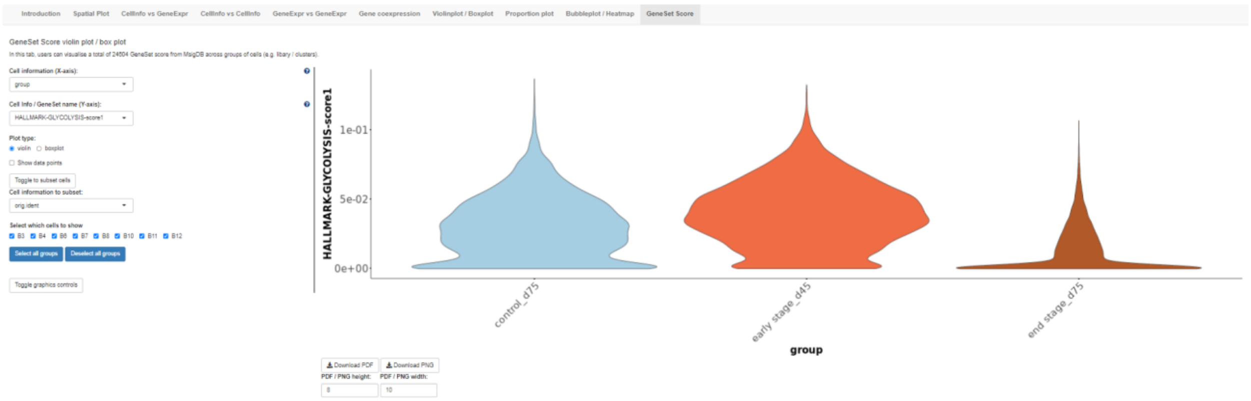

6.2.1. Cell Info/ Gene Set name (Y-axis):

- Use the dropdown menu under "Cell Info/ Gen Set name" to select a gene set/pathway of interest (e.g., "HALLMARK-GLYCOLYSIS-score1");
- Alternatively, you can type keywords related to your gene set of interest in the search box and select from the suggested options;
- Select Plot type for optimal visualization (Violin or boxplot);
- Select “Toggle to subset cells” to choose the cell information to subset; Choose between group or annotation to select specific brain regions;

6.3. Spatial geneset score: Spatial plot (on the spatial plot tab)

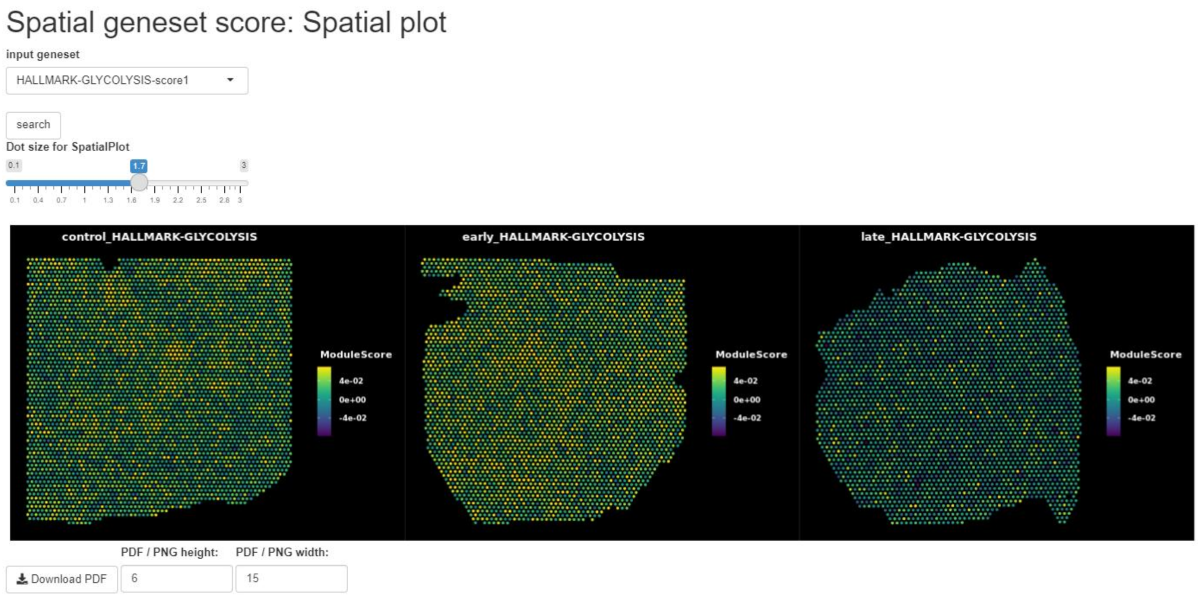

6.3.1. Input Gene Set:

- Use the dropdown menu under "input geneset" to select a gene set of interest (e.g., " HALLMARK-GLYCOLYSIS-score1”
- Alternatively, you can type keywords related to your gene set of interest in the search box and select from the suggested options.
- Click the "search" button to retrieve the gene set score data.

6.3.2. Spatial Plot:

- The spatial plot provides a visual representation of the gene set scores across different conditions and time points.
- Adjust the dot size for the spatial plot using the slider to change the visualization resolution.
- The example spatial plot displays the gene set score for " HALLMARK-GLYCOLYSIS-score1" across control, early and late PD conditions;
- Download the spatial plot as a PDF or PNG file by setting the desired dimensions and clicking the download buttons.
